# A conjugate self-organizing migration (CSOM) and reconciliate multi-agent Markov learning (RMML) based cyborg intelligence mechanism for smart city security

**DOI:** 10.1038/s41598-023-42257-0

**Published:** 2023-09-21

**Authors:** S. Shitharth, Abdulrhman M. Alshareef, Adil O. Khadidos, Khaled H. Alyoubi, Alaa O. Khadidos, Mueen Uddin

**Affiliations:** 1 Department of Computer Science, Kebri Dehar University, Kebri Dehar, Ethiopia; 2 Department of Information Systems, Faculty of Computing and Information Technology, King Abdulaziz University, Jeddah, Saudi Arabia; 3 Department of Information Technology, Faculty of Computing and Information Technology, King Abdulaziz University, Jeddah, Saudi Arabia; 4 Center of Research Excellence in Artificial Intelligence and Data Science, King Abdulaziz University, Jeddah, Saudi Arabia; 5 College of Computing and IT, University of Doha for Science and Technology, 24449 Doha, Qatar

**Keywords:** Engineering, Mathematics and computing

## Abstract

Ensuring the privacy and trustworthiness of smart city—Internet of Things (IoT) networks have recently remained the central problem. Cyborg intelligence is one of the most popular and advanced technologies suitable for securing smart city networks against cyber threats. Various machine learning and deep learning-based cyborg intelligence mechanisms have been developed to protect smart city networks by ensuring property, security, and privacy. However, it limits the critical problems of high time complexity, computational cost, difficulty to understand, and reduced level of security. Therefore, the proposed work intends to implement a group of novel methodologies for developing an effective Cyborg intelligence security model to secure smart city systems. Here, the Quantized Identical Data Imputation (QIDI) mechanism is implemented at first for data preprocessing and normalization. Then, the Conjugate Self-Organizing Migration (CSOM) optimization algorithm is deployed to select the most relevant features to train the classifier, which also supports increased detection accuracy. Moreover, the Reconciliate Multi-Agent Markov Learning (RMML) based classification algorithm is used to predict the intrusion with its appropriate classes. The original contribution of this work is to develop a novel Cyborg intelligence framework for protecting smart city networks from modern cyber-threats. In this system, a combination of unique and intelligent mechanisms are implemented to ensure the security of smart city networks. It includes QIDI for data filtering, CSOM for feature optimization and dimensionality reduction, and RMML for categorizing the type of intrusion. By using these methodologies, the overall attack detection performance and efficiency have been greatly increased in the proposed cyborg model. Here, the main reason of using CSOM methodology is to increase the learning speed and prediction performance of the classifier while detecting intrusions from the smart city networks. Moreover, the CSOM provides the optimized set of features for improving the training and testing operations of classifier with high accuracy and efficiency. Among other methodologies, the CSOM has the unique characteristics of increased searching efficiency, high convergence, and fast processing speed. During the evaluation, the different types of cyber-threat datasets are considered for testing and validation, and the results are compared with the recent state-of-the-art model approaches.

## Introduction

In recent days, the smart city technologies^1,2^ are developing rapidly due to the rise of global urbanization. As population density increases in urban areas, infrastructure and facilities are required to balance environmental and transportation issues. The smart cities^3–5^ are one of the most suitable option and solution for handling these problems. The development of smart city applications are highly depends on the factors of growth and low cost of Internet of Things (IoT) infrastructure^6,7^, which are integrated with the wireless communication technology. Typically, the IoT is one of the most essential component of smart city systems, because which interlinks the devices over the internet. In this application^8–10^, the data is collected and analyzed from the environment by using different physical sensors and wireless technologies. Moreover, the expansion of IoT in smart city networks require to ensure the properties of privacy, security, confidentiality, trust, scalability, and centralization. When compared to the other networking paradigms, the IoT systems^11–13^ are dissimilar and scattered in natured. Due to its capacity, bandwidth, and memory usage, the design and development of a trusted IoT system^14–16^ are the most challenging and crucial tasks. Also, the smart cities are highly susceptible to various cyber-threats, and is highly complex to manage the IoT devices in the network. Due to the rapid development of cloud systems, the storage complexity is efficiently resolved in recent times, because the cloud has an increased storage and computational power. Still, the number of challenges and security risks associated to the smart city technologies^17,18^ are exist. The Cyborg intelligence is one of the most suitable and perfect option for ensuring the safety and trustworthiness of the smart city networks. This technology helps to detect the threats in the network by analyzing its characteristics and monitoring the environment. In the conventional works, the different types of machine learning and deep learning based Cyborg intelligence techniques^19–22^ are developed for smart city security. However, it faced the problems and challenges correlated with the following terms^23–28^: complex to implement, high cost, not much capable to handle the large dimensional datasets, and more time for intrusion detection.

Global urbanization is increasing, which is leading to an increase in the prevalence of smart cities. As the population in metropolitan regions increases, infrastructure and facilities must deal with the ecological and transportation issues. The development of smart cities is a response to the aforementioned issues. The rapid growth and expansion of low-cost devices and other IoT-oriented infrastructure, which have been combined with wireless communication technology, have become increasingly important for the development of a wide range of smart city applications. Technology for smart cities includes the Internet of Things (IoT), which connects the computer devices (smart objects) over the Internet. In the technology, data is gathered and analyzed in almost real-time using a range of actual sensors and wireless connections. The information obtained from sensors is used to operate and process actuators. Therefore, in order to expand IoT infrastructure in smart cities, it will be necessary to guarantee key attributes including security, confidentiality, trust, flexibility, and centralization. IoT systems^29^ are less uniform and more varied than conventional systems. Therefore, in order to expand IoT infrastructure in smart cities, it will be necessary to guarantee key attributes including security, confidentiality, trust, flexibility, and centralization^30^. IoT systems are less uniform and more varied than conventional systems. The collected data needs to be secured against unauthorized access in order to guarantee the security and privacy of smart cities. In order to improve the quality of the people's daily lives, a smart city gathers and evaluates data about their health and the environment in which they live. There has been extensive research done on how to protect data from attackers, so it is not surprising. There are a few difficulties that must be resolved in order to promote the expansion of sustainable smart cities, which includes high system complexity, increased time consumption, and not-efficient.

*Onyema*, et al.^31^ developed a new IDS framework based on the Cyborg intelligence mechanisms for increasing the security of smart city networks. The purpose of this work was to highly enhance the security of IoT enabled networks by detecting an anomalous network traffic with the use of multiple algorithms. Typically, the privacy and security of the smart cities were maintained by protecting the collected data from the unauthorized activities. Moreover, an accurate and efficient Network Intrusion Detection System (NIDS) was developed in this work based on the context of Cyborg intelligence models. The key benefits of this work were better detection accuracy, and optimized performance rate. Yet, it has the major problems of increased training and testing complexity, which affects the efficacy of the suggested security framework. *Abosaq*, et al.^32^ investigated about the impacts privacy problems in the smart city networks. Typically, it is highly more essential to ensure the better communication and computational capabilities of the smart city networks for enabling an effective data transmission. Here, the different types of privacy issues associated to the smart city networks were discussed, which includes authentication, confidentiality, privacy, secrecy, and safety. *Mehra*, et al.^33^ objects to detect the ransomware attacks by developing an advanced cyber-security framework. *Priyadharshini*, et al.^34^ implemented a Merkle-based security mechanism for securely allocating the users in the distributed environment. Also, an identity based authentication mechanism was utilized in this work in order to authorize the users for enabling a secured data communication. However, this work does not has the ability to handle the large dimensional IDS datasets, which was the major limitation of this work. *Thiyagarajan*, et al.^35^ conducted a detailed review for analyzing various machine learning and deep learning techniques used for developing an efficient cybersecurity framework. The scope of this paper was to investigate the efficiency and performance of the Artificial Intelligence (AI) mechanisms for ensuring a complete information security. However, it failed to validate the performance of the machine learning/deep learning techniques, hence it is difficult to identify the most suitable mechanism.

*Alazzam*, et al.^36^ employed a Pigeon Inspired Optimization (PIO) technique for designing an accurate IDS with reduced computational complexity. The contribution of this paper was to utilize the feature selection algorithm for optimizing the performance of classifier with increased convergence speed and minimal time consumption. Here, the detection performance of this IDS framework was validated and testing by using various network datasets such as DARPA, UNSW-NB15 and NSL-KDD. However, it does not utilize an efficient classifier for accurately predicting the type of intrusion, which was the major limitation of this work. *Salloum*, et al.^37^ presented a comprehensive literature review for validating the different types of machine learning and deep learning techniques used for detecting the normal and attacking activities in the network. Moreover, it used the different types of cybersecurity datasets for analyzing the efficiency of the ML/DL techniques, which includes the Information Security and Object Technology (ISOT) dataset, HTTP CSIC 2010, Czech Technical University (CTU-13), and UNSW-NB 15. Based on this review, it is analyzed that the AI mechanisms could be more useful for developing an effective security systems. *Hindy*, et al.^38^ deployed a machine learning based IDS framework for ensuring the security of IoT networks. In this system, the MQTT-IoT-IDS2020 dataset has been utilized to test the performance of this system. The purpose of this work was to categorize the normal and benign traffic by using 6 different types of machine learning techniques. According to this analysis, it is studied that the DT technique outperforms the other approaches with increased detection results.

*Duraisamy*, et al.^39^ implemented a Krill-Herd (KH) optimization integrated Deep Learning Neural Network (DLNN) technique for improving the security of smart city networks. The KH was one of the most popular optimization technique extensively used for feature selection and dimensionality reduction. In addition to that, the min-max normalization mechanism was utilized to preprocess the given dataset. The key benefits of this work were increased detection accuracy, high level of security, and minimal time consumption. However, it has the following limitations: difficult to understand, reduced convergence rate, and complex mathematical calculations. *Alsarhan*, et al.^40^ deployed a Support Vector Machine (SVM) classification technique for detecting intrusions in the Vehicular Ad-hoc Networks (VANETs). Here, three different types of optimization techniques such as Particle Swarm Optimization (PSO), Ant Colony Optimization (ACO), and Genetic Algorithm (GA) were separately used for selecting the most suitable technique. From this study, it is analyzed that the combination GA-SVM outperforms the other approaches with increased performance results. Also, it has the key benefits of reduced false positives, error rate, and better convergence speed.

Reference^41^ integrated the federated learning model with the smart city application systems for improving its security and privacy. The focus of this work is to conduct a comprehensive review for analyzing the list of AIoT techniques for maximizing the network security. *Bangui*, et al.^42^ presented a comprehensive survey for investigating the recent machine learning techniques used to develop an advanced IDS framework. It includes the popular mechanisms of Recurrent Neural Network (RNN), Game Theory, SVM, K-means, Self- Organizing Map (SOM), Logistic Regression (LR), and Random Forest (RF). Among other mechanisms, the RNN provides an increased detection accuracy and efficiency. *Maseleno*, et al.^43^ deployed a Random Monarch Butterfly (RMB) optimization integrated RNN technique for protecting the smart society networks against the cyber-threats. During optimization, the migration and butterfly adjusting operators have been used to identify the best optimal solution with reduced number of iterations. Moreover, the attack detection performance of this system was validated and tested according to the parameters of detection level, f-measure, accuracy, and error rate. The primary advantages of this technique were capability of handling large dimensional datasets, reduced training and testing time. Table 1 reviews some of the recent state-of-the-art model techniques used for smart city security and intrusion detection^44^.

**Table 1:** Recent state-of-the-art model analysis.

References	State of the art methods	Descriptions	Findings
^45^	Kernel based Principal Component Analysis (PCA)	In this work, an AI based PCA model is implemented for identifying intrusions from smart city-IoT networks	Better performance outcomes, low false prediction, and high system complexity
^46^	KNN based intrusion detection model	It aims to improve the security while exchanging data in smart city networks	Low accuracy, and not suitable for handling large dimensional data
^47^	Cluster enabled Multi-task learning model	It aims to handle different types of attacks in a mobile crowd sourcing environment	Ensured Quality of service, and better efficiency in attack detection
^48^	Federated learning mechanism	In this study, an ensemble weighted average approach has been used to categorize the normal and intrusive events from the smart city networks	Computational complexity and overfitting
^49^	Machine learning model	Here, the different types of machine learning algorithms are implemented for intrusion detection	High reliability, fast in process, and better prediction rate

The motivations behind the proposed are given below:

- To thoroughly investigate the research gaps in the linked devices of smart cities' network intrusion detection procedure.
- To create a network intrusion detection system for smart cities that is more precise and effective.
- Developing the aforementioned mechanism in the framework of cyborg intelligence to gain from both machine and human intelligence.
- To put into practice the proposed method of a specific dataset for evaluating the effectiveness of this mechanism.
- To evaluate the success rate of current machine learning techniques for network intrusion detection.

Therefore, the proposed work intends to develop a novel Cyborg intelligence mechanism for securing the smart city networks with reduced computational and time complexity. The major research objectives of this paper are as follows:

- To design and develop a novel Cyborg intelligence based security model for protecting the smart city networks against the cyber-threats.
- To normalize and preprocess the input cyber-threat datasets, the Quantized Identical Data Imputation (QIDI) mechanism is employed that effectively improves the quality of dataset by filtering the attributes.
- To optimally choose the features for training the classifier model, an intelligent and advanced Conjugate Self-Organizing Migration (CSOM) based optimization algorithm is developed.
- To accurately predict the intrusion with its category, a novel Reconciliate Multi-Agent Markov Learning (RMML) based classification approach is implemented.
- To test and validate the results and efficacy of the proposed CSOM-RMML mechanism, the different types of evaluation indicators are estimated.

The other portions of this paper are organized into the followings: Section “Methods” presents the clear description about the proposed CSOM-RMML based Cyborg intelligence mechanism with its appropriate working flow and algorithms. The results of the proposed mechanism are validated and compared by using different datasets and parameters in Section “Results”. At last, the entire paper is summarized with its findings, challenges, and future work in Section “Conclusion”.

## Methods

**Figure 1:**
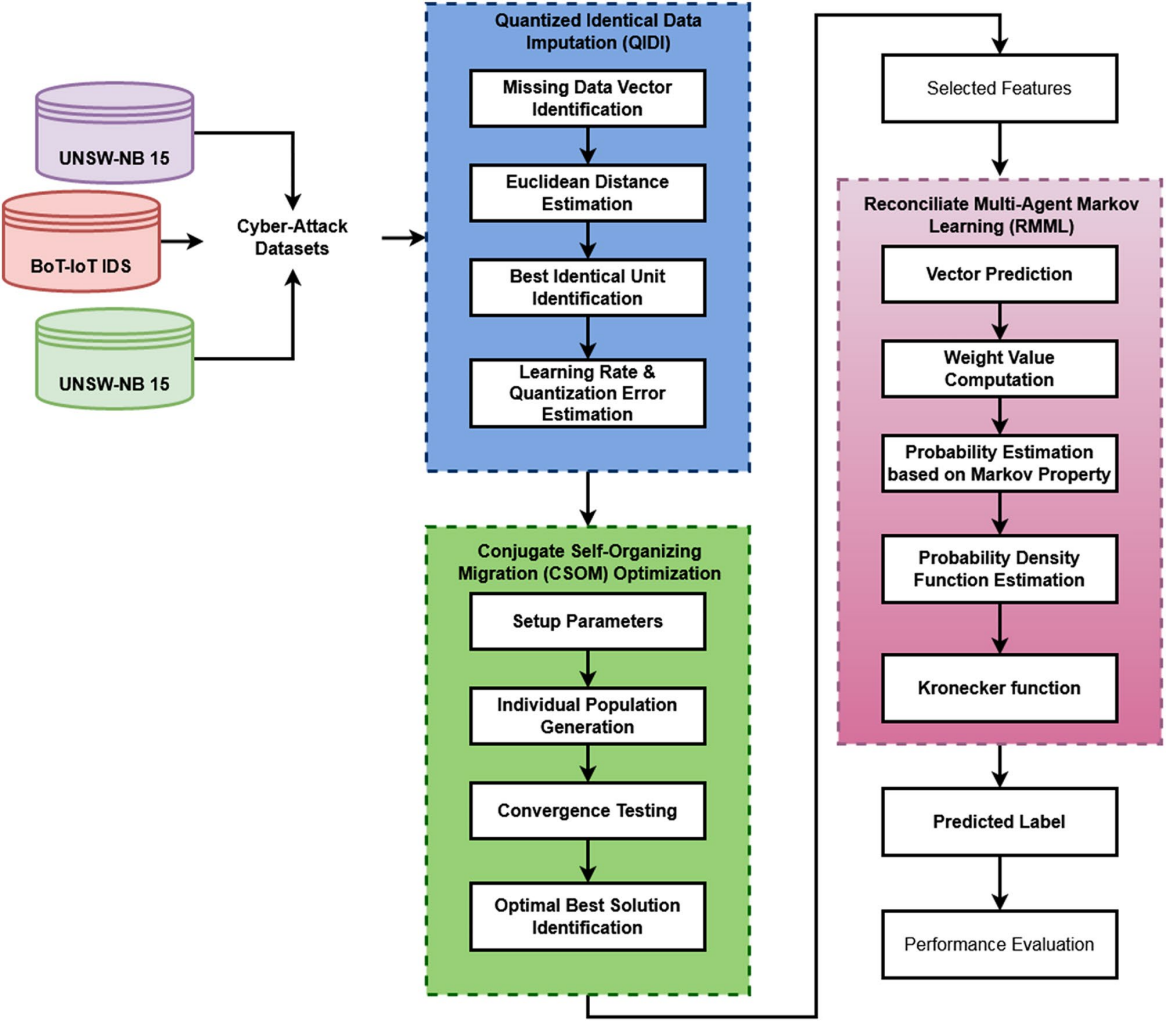
Working flow of the proposed Cyborg intelligence based security framework.

This section presents the clear description about the proposed Cyborg intelligence model for increasing the security of smart city systems. The original contribution of this work is to implement a novel optimization and classification techniques for designing a novel IDS framework to protect the smart city networks against the cyber-threats. The overall working flow of the proposed system is shown in Fig. 1, which includes the following stages:

- Dataset Preprocessing and imputation
- Feature Optimization
- Intrusion Classification

Here, the different types of cyber-attack datasets are taken as the inputs for processing, which includes the UNSW-NB15, DS2OS, CICIDS-2017, BoT-IoTIDS 2020, and NSL-KDD. These are all the most popular benchmark datasets highly used in many application systems. At first, the Quantized Identical Data Imputation (QIDI) is used to preprocess the cyber-attack datasets by identifying the missing data vectors. Also, it helps to improve the overall quality of input for obtaining the maximum detection performance. After that, the Conjugate Self-Organizing Migration (CSOM) based optimization algorithm is implemented to choose the optimal number of features for classifier training and testing. The primary purpose of using this mechanism is to accurately detect the cyber-threats with increased computational efficiency. Consequently, the Reconciliate Multi-Agent Markov Learning (RMML) based classification methodology is used to predict and categorize the type of cyber-threat against the smart city networks. The key benefits of using the proposed CSOM-RMML based Cyborg intelligence mechanism are high security level, increased attack detection performance, easy to understand, and minimal complexity in computations.

### Preprocessing

For different types of data, different cleaning techniques are required. In machine learning, missing data must be treated with caution since it is more essential. There are two approaches to handle missing data, although both may produce information that is less than ideal:

- Eliminating records with values that are missing: This is not the best course of action because it could result in the loss of information that could be instructive.
- Using previous observations to impute the missing values: It is not ideal and could result in information loss because the value was initially missing but we added it.

In this stage, Quantized Identical Data Imputation (QIDI) mechanism is employed to preprocess the given cyber-threat datasets by normalizing the attributes. Here, the main purpose of using this preprocessing technique is to increase the quality of data by identifying the missing fields, and eliminating the irrelevant attributes. This preprocessed data helps to obtain an improved classification performance. Conventionally, the different types of filtering and normalization techniques are used in the existing works for dataset preprocessing. However, it has the problems of presence of noise, inconsistency, and error values. Therefore, the proposed work objects to employ a new QIDI mechanism for preprocessing the datasets, which has the key benefits of simple to understand, easy to implement, less time for processing, and high quality of data. During this process, the missing input data vector is generated at first, as shown in below:

1 $$X=\left[{x}_{1},{x}_{2}, \dots , {x}_{i} \right]; i=\mathrm{1,2},\dots , N,$$

where, N indicates the number of features. After that, the distance is estimated for the individual features according to the weight vector by using the following equation:

2 $${D}_{i}= \sqrt{\sum_{k=1}^{n}{m}_{k} {\left({x}_{k}-{w}_{ik} \right)}^{2} },$$

where, ${D}_{i}$ indicates the Euclidian distance between the input vector and weight vector *i*, ${x}_{k}$ = $k$ is the element of current vector, *n* indicates the dimension of the input vector and ${w}_{ik}$= *k* denotes the element of the weight vector *i*. Consequently, the mask value is estimated based on the input vector as shown in below:

3 $${m}_{k}= \left\{\begin{array}{ll}0& \quad Input \,vector \,contains\, 0\, for \,each\, column\\ 1& \quad Otherwise\end{array}\right. ,$$

where, ${m}_{k}$ denotes the mask value. Then, the Best Identical Unit (BIU) is identified by adjusting the weight vector of the winner neuron. Hence, the BIU and its adjacent neurons are move closer to the input vectors in the space, which also helps to increase the agreement between the input and weight vectors. This adjustment is carried out by using the following model:

4 $${w}_{t} \left(t+1\right)={w}_{t}\left(t\right)+\eta \left(t\right) {h}_{f} \left[x\left(t\right)-{w}_{t}\left(t\right)\right],$$

where, ${w}_{t}$ indicates the element of weight vector, $t$ is the time factor, $\eta \left(t\right)$ is the learning rate, and ${h}_{f}$ is the neighborhood function. Consequently, the learning rate monotonically decreases with the number of iterations increased as represented in the following model:

5 $$\eta \left(t\right)= {\eta }_{0} {\left(\frac{0.005}{{\eta }_{0}}\right)}^{t/T},$$

where, ${\eta }_{0}$ initial learning rate and $T$ training length. After that, the quantization error is estimated by using the following model:

6 $${E}_{q}=\frac{1}{N} \sum_{i=1}^{N}\Vert {X}_{i}-{W}_{ib}\Vert ,$$

where, $N$ is the number of input vectors used to train the map, ${W}_{ib}$ prototype weight vector of the best matching unit of ${X}_{i}$, and $\Vert .\Vert$ denotes the Euclidean distance. Finally, the proportion of variance of a variable is predicted from other variable by using the following equation:

7 $$p{r}_{var}=\frac{\sum_{i=1}^{n}[({x}_{i}-{x}{^\prime})({y}_{i}-{y}{^\prime})]}{\sum_{i=1}^{n}\left[{\left({x}_{i}-{x}{^\prime}\right)}^{2}\right] \sum_{i=1}^{n}\left[{\left({y}_{i}-{y}{^\prime}\right)}^{2}\right]},$$

where, ${x}_{i}$ indicates the observed value of ${i}^{th}$ features, ${y}_{i}$ represents the trained value of ${i}^{th}$ features, ${x}{^\prime}$ denotes the mean of observed value,${y}{\prime}$ is the mean of the trained value, and n represents the number of observations. Based on this process, the given input cyber-threat dataset is preprocessed and the attributes are normalized.


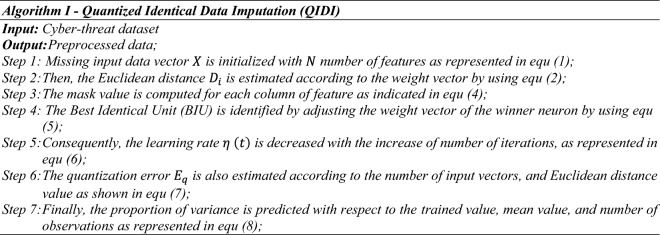


### Feature optimization

Furthermore, we employed a feature optimization approach to reduce the input dimension through choosing the optimal feature subset. After imputation, the Conjugate Self-Organizing and Migration (CSOM) optimization algorithm is employed to optimally choose the features for classifier training and testing. In the existing smart city frameworks, various nature-inspired and bio-inspired optimization techniques are utilized for reducing the dimensionality of data and, improving the detection rate of classifier. Nevertheless, they have the major problems of reduced convergence speed, more number of iterations for reaching the optimal solution, high time consumption, and complex computations. Thus, the proposed work motivates to develop a novel and intelligent optimization technique for selecting the relevant features from the normalized cyber-threat datasets. It is motivated by the smart, successful, and cooperative behavior of population members who use numerous migration loops to find the problem's ideal, world-wide solution. A stochastic optimization technique that draws inspiration from the intelligence of creatures like birds and fish. The goal of the field of numerical optimization is to look for globally optimal solutions. In order to do that, the technique starts by creating a population of a certain number of people, each of whom is a potential solution to the issue. Through numerous migration loops, further solutions that were superior to the first ones based on rivalry and collaboration amongst these individuals, a crucial component of the swarm intelligent algorithm, are subsequently produced. Then it is continued until the algorithm's specified stop criteria are met. This mechanism encompasses the following operations:

- Parameter initialization
- Individual population generation
- Convergence testing
- Refinement
- Best optimal solution identification

### Setup parameters

At first, the setup parameters are initialized that includes the controlling parameters $Nu{m}_{set}$, $CPT$, $po{p}_{no}$, stopping parameters $Mig$, $Dis{t}_{m}$, and iteration count $cn{t}_{m}$.

- $Nu{m}_{set}$ is a controlling parameter that defines the number of steps before the end of the movement.
- $CPT$ is an another controlling parameter that determines that whether the individual population will move along the chosen coordinate to the leader. The possible value is 0.3;
- $po{p}_{no}$ is a control parameter that is used to estimate the size of the individual population. Suggested value $po{p}_{no}$ > 10;
- Migration ($Mig$) is a stopping parameter showing the maximum number of iterations. Suggested $Mig$ > 10;
- $Dis{t}_{m}$ is a stopping parameter that is determined based on the value of goal function, which duplicates the average deviation among the three population leaders. The algorithm will come to a halt if this number is less than the target value. Once the value is entered, the condition is verified as, if it is negative, the condition won't be satisfied, and the search will end once the allotted number of migration cycles has been reached.
- $cn{t}_{m}$ is an iteration counter required to terminate the algorithm when it reaches the Migration number.
- Let $$cn{t}_{m}$$ =0;

### Generation of individual population

After that, the individual population is generated according to the coordinates, which are randomly generated ${x}_{j}$ within the interval of $\left[{\alpha }_{j},{\beta }_{j}\right]$ as represented below:

8 $${x}_{j}={\alpha }_{j}+ran{d}_{j}\left[\mathrm{0,1}\right]\left({\beta }_{j}-{\alpha }_{j}\right), j=\mathrm{1,2},\dots ,n,$$

where, $n$ is the number of iteration.

### Migration loop

Consequently, the migration loop is executed, in which the leaders are selected at first according the best values. This selection is carried out after evaluating each individual by using the objective function. During this process, the population is sorted according to the target function in a non-decreasing order $\{{x}_{1},\dots ,{x}_{po{p}_{no}}\}$. After that, the first three individuals are selected with respect to the lowest value of the objective function. For all individuals, two clones are created, which is an individual with the same coordinated as represented in below:

$$for \,all\, k=1,\dots ,po{p}_{no}$$

9 $${x}_{j}^{po{p}_{no}+k }={x}_{j}^{k}$$

10 $${x}_{j}^{2*po{p}_{no}+k }={x}_{j}^{k}; j=\mathrm{1,2},\dots ,n.$$

End for;

Moreover, the random number is created for each individual’s coordinates, before they begin to travel in the direction of leader. Then, it is compared to the controlling parameter CPT as represented in below:

11 $$CPTvecto{r}_{j}=\left\{\begin{array}{l}1\, \quad if \,ran{d}_{j}<CPT\\ 0 \, \quad otherwise\end{array}\right. \quad j=\mathrm{1,2},\dots ,n.$$

Subsequently, all other individuals starts to move towards the leader, where the movement occurs in steps until the final destination on the iteration is reached with respect to the parameter $Nu{m}_{set}$. It is estimated for the first leader by using the following model:

12 $${x}^{k,t}={x}^{k}+\frac{({x}^{1}-{x}^{k})}{2*Nu{m}_{set}}*{t}_{1}* CPTvecto{r}_{j}$$

13 $${t}_{1}=\mathrm{0,1},\dots ,4*Nu{m}_{set}$$

14 $$k=\mathrm{1,2},\dots , po{p}_{no}.$$

Consequently, it is also estimated for the second and third leaders by using the following models:

15 $${x}^{k,t}={x}^{k}+\frac{({x}^{2}-{x}^{k})}{Nu{m}_{set}}*{t}_{2}* CPTvecto{r}_{j}$$

16 $${t}_{2}=\mathrm{0,1},\dots ,2*Nu{m}_{set}$$

17 $$k=\left(po{p}_{no}+1\right),\dots , (2*po{p}_{no})$$

18 $${x}^{k,t}={x}^{k}+\frac{({x}^{3}-{x}^{k})}{\left[\frac{Nu{m}_{set}}{2}\right]}*{t}_{3}* CPTvecto{r}_{j}$$

19 $${t}_{3}=\mathrm{0,1},\dots ,2*Nu{m}_{set}$$

20 $$k=\left(2*po{p}_{no}+1\right),\dots , \left(3*po{p}_{no}\right).$$

After all movements, the best step is identified for each individual (the step at which the value of the objective function is small), and the individual takes this location, assigning itself with the corresponding coordinate values, and moves to the next population. It is estimated for the first leader as shown in below:

21 $${x}^{k,New}=\underset{{t}_{1}=\mathrm{0,1},\dots ,4*\mathit{Nu}{m}_{\mathit{set}} }{\mathit{arg}\mathrm{min}}f({x}^{k, {t}_{1}})$$

22 $$k=\mathrm{1,2},\dots , po{p}_{no}.$$

For the second and third leaders, the functions are calculated as follows:

23 $${x}^{k,New}=\underset{{t}_{2}=\mathrm{0,1},\dots ,2*\mathit{Nu}{m}_{\mathit{set}} }{\mathit{arg}\mathrm{min}}f\left({x}^{k, {t}_{2}}\right)$$

24 $$k=\left(po{p}_{no}+1\right),\dots , (2*po{p}_{no})$$

25 $${x}^{k,New}=\underset{{t}_{3}=\mathrm{0,1},\dots ,\mathit{ }\mathit{Nu}{m}_{\mathit{set}} }{\mathit{arg}\mathrm{min}}f({x}^{k, {t}_{3}})$$

26 $$k=\left(2*po{p}_{no}+1\right),\dots , (3*po{p}_{no})$$

#### Convergence testing

Furthermore, the convergence speed of this optimization algorithm is validated by using the following model:

If $$\sqrt{\frac{1}{2}\sum_{k=2}^{3}{\left[f\left({x}^{k}\right)-f\left({x}^{1}\right)\right]}^{2}} \ge Dis{t}_{m}$$ and $$cn{t}_{m}<Mig.$$

If the above conditions are satisfied, the maximum number of migrations are not reached, then go to Step A; Otherwise, go to Step B:

Step A:

- Updating the population.
- Sort all individuals based on the non-decreasing objective function:
  27 $$\left\{{x}^{1},\dots , {x}^{P}\right|f({x}^{p})\le f({x}^{p+1}))\} , p=1,\dots ,P-1$$

Where $$P=3*po{p}_{no}$$;

- Remove the last $2*po{p}_{no}+\left[\frac{1}{3}*po{p}_{no}\right]$ individuals thus leaving only $\left\{{x}^{1},\dots , {x}^{\left[\frac{2}{3}*po{p}_{no}\right]}\right\}$ .
- Generate $\left[\frac{1}{3}*po{p}_{no}\right]$ new individuals,
  28 $${x}_{j}^{k}= {\alpha }_{j}+ran{d}_{j}\left[\mathrm{0,1}\right] \left({\beta }_{j}-{\alpha }_{j}\right)$$
  where, $$k=\left[\frac{2}{3}*po{p}_{no}\right], \dots ,po{p}_{no}$$ and $$j=\mathrm{1,2},\dots ,n$$
- Increase the iteration counter:
  29 $$cn{t}_{m}= cn{t}_{m}+1$$

Step B:

- Refinement and stop criterion of algorithm;
- Increase the $Nu{m}_{set}$ parameter and conduct a migration cycle for the second and third leaders relative to the first leader:
  30 $$Nu{m}_{set}=10*Nu{m}_{set}$$
  31 $${x}^{k,t}={x}^{k}+\frac{\left({x}^{1}-{x}^{k}\right)}{2*Nu{m}_{set}}*t* CPTvector$$
  $$k=\mathrm{1,2},3$$

Predict new leader position,

32 $${x}^{k,New}=\underset{t=\mathrm{0,1},\dots ,\mathit{ }\mathit{Nu}{m}_{\mathit{set}} }{\mathit{arg}\mathrm{min}}f({x}^{k,t}) k=\mathrm{1,2},3$$

Returning the best solution found during the search:

33 $${x}^{pred}=\underset{k=\mathrm{1,2},3 }{\mathit{arg}\mathrm{min}}f({x}^{k})$$

Based on the best solution, the features are optimally selected for training the classifier.

### Reconciliate multi-agent Markov learning (RMML)

After feature selection, the novel technique, named as, Reconciliate Multi-Agent Markov Learning (RMML) is employed to predict and categorize the intrusion according to the selected features. It is a kind of machine learning mechanism mainly used for accurately predicting the cyber-threats against the smart city networks. In the existing works, the different types of machine learning techniques such as DT, RF, LR, SVM, KNN, and etc. are developed for developing an effective IDS security framework. Yet, it has the drawbacks of increased false alarm rate, error rate, complex to understand, high training and testing time. Hence, the proposed work intends to develop an advanced Cyborg intelligence mechanism by designing an optimization incorporated machine learning classification methodology, which helps to ensure the security of smart city networks against the cyber-threats. In the proposed work, the RMML based machine learning model is mainly used to predict the intrusion from the smart city networks. This algorithm is developed based on the conventional multi-agent markov decision technique, which is more suitable for handling the prediction problems. When compared to the other machine learning techniques, the proposed RMML has the primary advantages of low computational complexity, reduced time consumption, and high training speed. In the proposed technique, the probability density function is estimated for taking an accurate decisions while predicting intrusions from the intrusion data. Typically, the deep learning techniques consume more time for training and testing data samples, and also it follows some complex computational operations to obtain the best classification results. When comparing to the deep learning techniques, the machine learning techniques consume less time to provide the classified label. But, their accuracy and efficiency were not up to the mark. Hence, the proposed work aims to implement the novel and effective machine learning technique for intrusion identification and classification. In this model, the vector prediction, weight matrix formulation, coupling coefficient estimation, and probability density function estimation are performed to take an accurate decisions at the time of intrusion detection, which lowers the complexity of classification with ensured accuracy.

Initially, the samples in ${i}^{th}$ label is represented as ${u}_{i}$, and samples for the ${j}^{th}$ is considered as ${s}_{j}$. After that, the vector prediction is performed by inferring all features in the layer *i* as represented in below:

34 $${u}_{j|i}={W}_{ij}{u}_{i}+{B}_{j}$$

where, ${W}_{ij}$ is the transformation matrix which is connected to decision process, ${u}_{i}$ be the prediction vector for ${i}^{th}$ label, and ${B}_{j}$ indicates the bias of the ${j}^{th}$ label. After that, the prediction vector for the ${j}^{th}$ label is considered as vote, and the weight matrix is estimated according to the coupling coefficient as shown in below:

35 $${s}_{j}=\sum_{i}{Z}_{ij}{u}_{j|i},$$

where, ${Z}_{ij}$ is the dynamic coupling coefficient that is computed as follows:

36 $${Z}_{ij}=\frac{\mathrm{exp}({p}_{ij})}{\sum_{j}\mathrm{exp}({p}_{ij})},$$

where, ${p}_{ij}$ indicates the probability that common features between ${i}^{th}$ label and ${j}^{th}$ label. Consequently, the probability value is computed for each category of label by using the following equation:

37 $${\delta }_{j}= \frac{{\Vert {s}_{j}\Vert }^{2}}{1+ {\Vert {s}_{j}\Vert }^{2}} * \frac{{s}_{j}}{\Vert {s}_{j}\Vert },$$

where, ${\delta }_{j}$ obtained from voting is computed by the multiple iterations of the algorithm model training to update ${p}_{ij}$. Moreover, the backpropagation function is used to optimize the network parameters with the interval of loss function as represented in below:

38 $$Los{s}_{c}=\sum_{c=1}^{Clas{s}_{no}}({I}_{c} \mathrm{max}(0,{m}^{+}-\Vert {\delta }_{c}\Vert ))+ \vartheta \left(1-{I}_{c}\right){\mathrm{max}\left(0,\Vert {\delta }_{c}\Vert -{m}^{-}\right)}^{2},$$

where, $c$ is the number of categories of the training samples, and ${I}_{c}$ is the indicator function as calculated below:

39 $${I}_{c}=\left\{\begin{array}{l}1 \quad if \,c\, is\, exist \,in \,samples\\ 0 \,does \,not\end{array}\right.,$$

where, ${m}^{+}$ indicates the upper bound correcting false positives, ${m}^{-}$ denotes the upper bound correcting false negatives, $\vartheta$ is the sale factor that adjust both upper and lower bounds, and $clas{s}_{no}$ is the total number of class. Based on the markov property and theory of probability of moving estimation, the discriminant for the sample corresponding to the category is estimated by using the following model:

40 $$\mathrm{Pr}\left({y}{\prime}|X\right)=argmax\mathrm{Pr}\left(Y|X\right) =argmax\frac{\mathrm{Pr}\left(X|Y\right)\mathrm{Pr}(Y)}{\mathrm{Pr}(X)},$$

where, $\mathrm{Pr}\left(X|Y\right)$ is the probability density function of the data, $\mathrm{Pr}(X)$ is the prior probability distribution of the each data of the particular category, and $\mathrm{Pr}(Y)$ is the prior probability distribution of the each data of the any category. Then, the markov theory is computed in below:

41 $$\mathrm{Pr}\left(Y\right)=\frac{1}{S}\mathrm{exp}\left\{-\tau \sum_{c \in C}{f}_{c}\left(x\right)\right\},$$

where, $c$ denotes the sub category of each label, $C$ indicates the number of class, and $\tau$ controlling parameter of the space term as represented in below:

42 $$S=\sum_{Y}\mathrm{exp}\{-R(Y)\},$$

where, $R$ is the random field of the normalization constant, and the potential function ${f}_{c}(x)$ is computed as follows:

43 $${f}_{c}\left(x\right)=\left\{\begin{array}{l}-1\, \quad if \,sample\, belongs\,to\, same\, category\\ +1 \,otherwise\end{array}\right..$$

Furthermore, the probability density function is estimated as represented in below:

44 $$\mathrm{Pr}\left(X|Y\right)= \prod_{i=1}^{M}p\left({x}_{i}\right|{y}_{i})= \prod_{i=1}^{M}\frac{\mathrm{Pr}\left({y}_{i} \right| {x}_{i}) \mathrm{Pr}({x}_{i})}{\mathrm{Pr}({y}_{i})},$$

where, ${x}_{i}$ is the ${i}^{th}$ sample, ${y}_{i}$ is the category of the ${i}^{th}$ sample and $M$ is the total number of samples. Based on the Markov decision formula, the probability is obtained for the category of,

45 $$\mathrm{Pr}\left(X|Y\right)\propto \prod_{i=1}^{M}\frac{p\left({x}_{i}\right|{y}_{i})}{p\left({y}_{i}\right)}P\left(Y\right)\propto \prod_{i=1}^{M}\frac{\mathrm{Pr}\left({y}_{i} \right| {x}_{i}) }{\mathrm{Pr}\left({y}_{i}\right)}\mathrm{exp}\left\{-\tau \sum_{c \in C}{f}_{c}\left(x\right)\right\}.$$

Then, this probability function is converted into a negative algorithmic form, where the problem of probability maximization is transformed into the minimum value problem by using the following model:

46 $$R\left({x}_{i}\right)=\mathrm{lnPr}\left(Y|X\right) = \sum_{i=1}^{M}\left[\mathrm{lnPr}\left({y}_{i}|{x}_{i}\right)-\mathrm{lnPr}\left({y}_{i}\right)\right]+ \sum_{{x}_{i} \in {M}_{i} }\tau \left(1-\partial \left({y}_{i},{y}_{i}{\prime}\right)\right),$$

where, $\left(a,b\right)$ is the Kronecker function, $\mathrm{Pr}\left({y}_{i}|{x}_{i}\right)$ is the posterior probability of the output value from the neural network, and $\mathrm{Pr}\left({y}_{i}\right)$ is the prior probability of the category. It is calculated based on the proportion of the current category after each iteration and, used as the input value for the next iteration. Based on this model, the proposed classifier predicts and categorizes the type of cyber-threat with reduced training and testing time.

## Results

This section validates and compares the performance and results of the proposed Cyborg intelligence mechanism used for ensuring the security of smart city networks. To test this security systems, the different types of cyber-threat datasets are utilized in this work, which includes the UNSW-NB 15, NSL-KDD, BoT-IoT IDS, DS2OS, and NSL-KDD. Moreover, the performance of the proposed optimization technique is also validated according to the number of iterations, best score, objective space, average fitness value, and searching history. In this study, the three distinct and well-known datasets UNSW-NB 15, BoT-IoT, and DS2OS are used for verifying and evaluating the proposed CSOM-RMML approach. Since these datasets are among the most recent and widely used in security application systems, they are also the most recent and popular public datasets. Additionally, it contains contemporary assaulting data that could be quite helpful for analyzing network attacks. These datasets are used by the proposed system to assess the performance and results of the system because of their emergence, popularity, and ease of accessible. Additionally, ToN-IoT, another current dataset, is employed in this study to assess the superiority of the suggested work. For large-scale application contexts like smart cities, IoT, IIoT, and others, the suggested datasets are appropriate. The results show that the suggested CSOM-RMML could handle these datasets with excellent accuracy and efficacy. As a result, it can handle very large intrusion datasets with superior performance and prediction rate.

**Figure 2:**
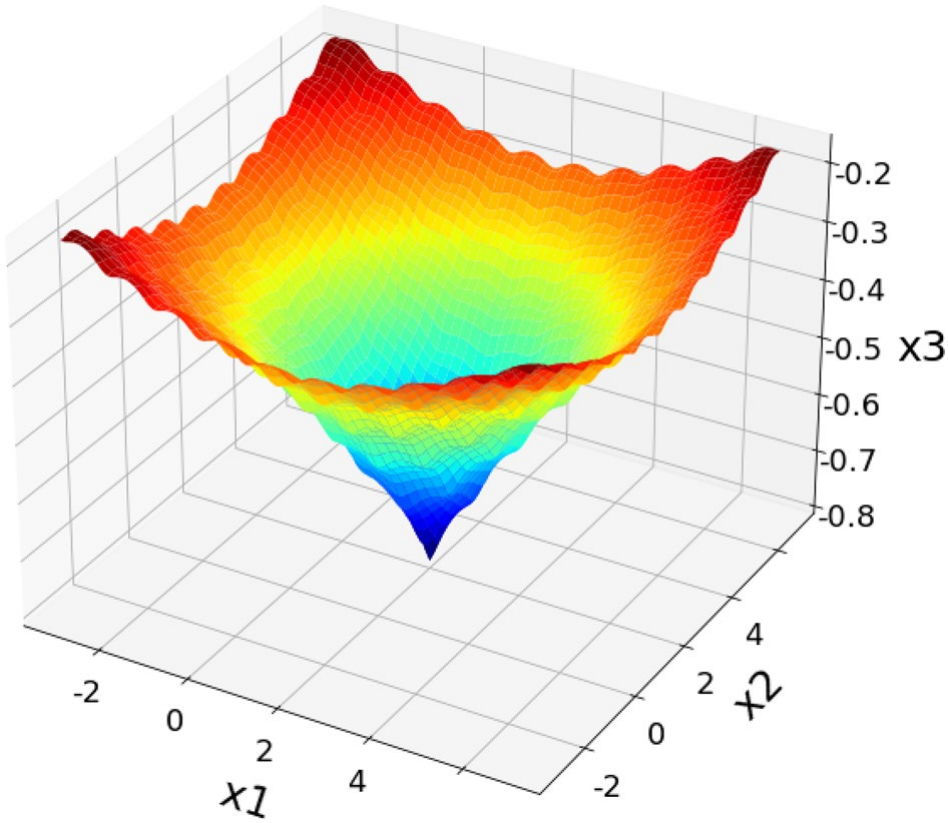
Benchmark testing.

**Figure 3:**
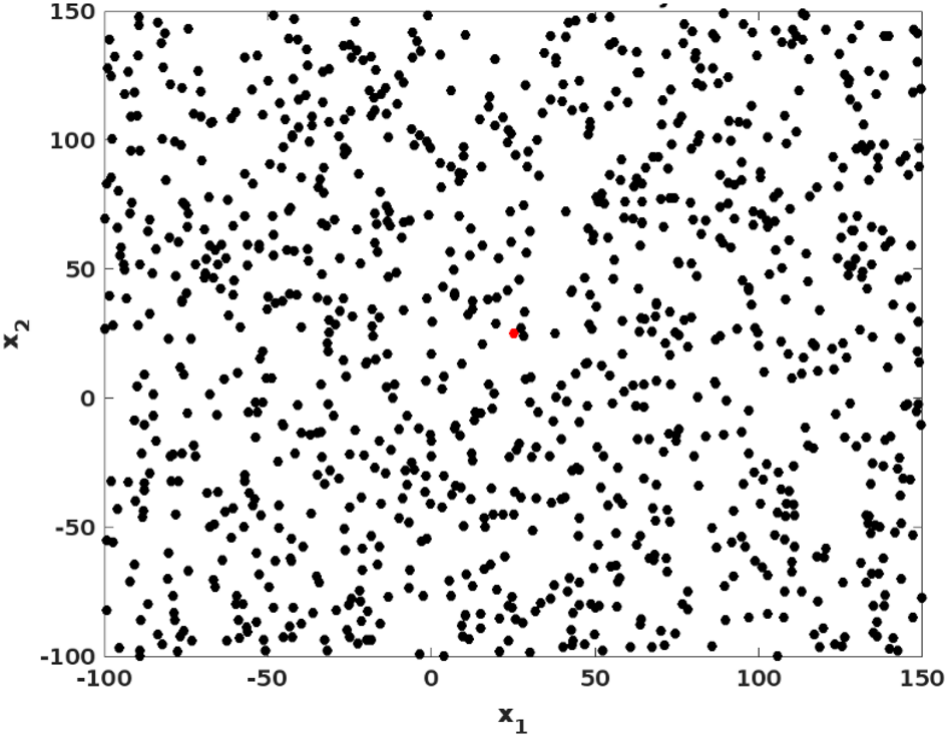
Searching history.

**Figure 4:**
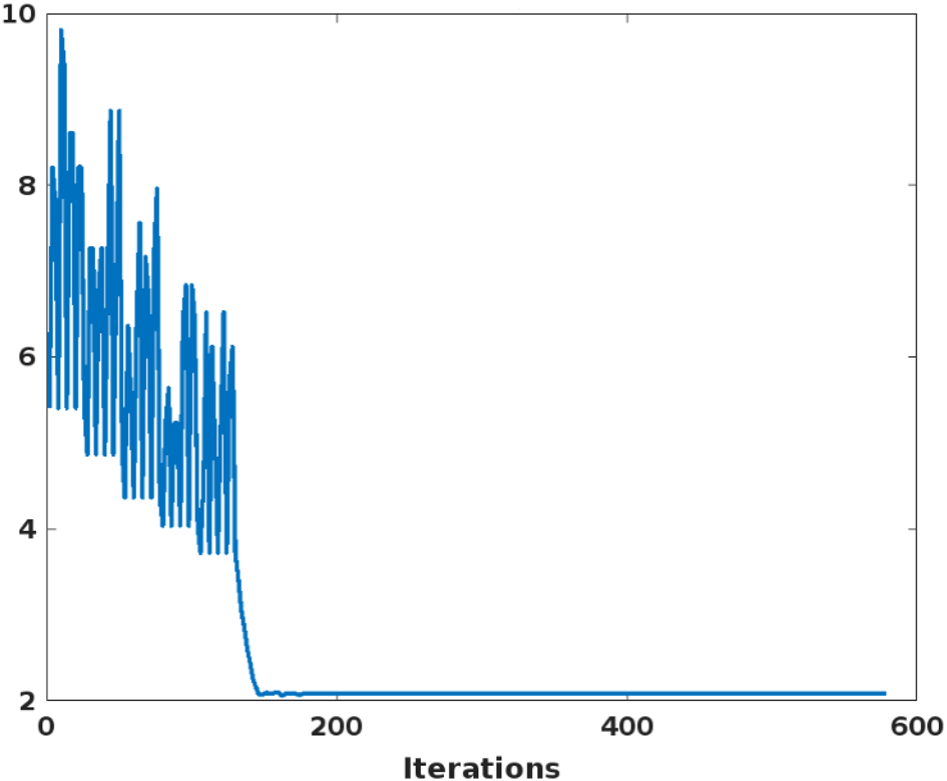
Average fitness value.

**Figure 5:**
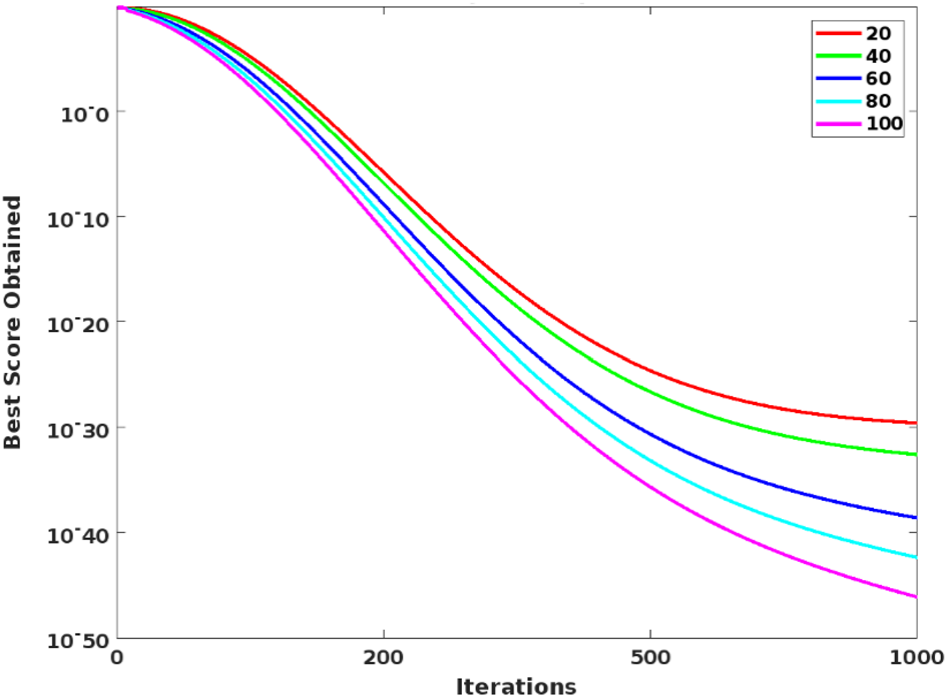
Best score Vs Number of iterations.

Figure 2 shows the estimated benchmark testing function of the proposed CSOM optimization technique, then its corresponding searching history and average fitness value are shown in Figs. 3 and 4 respectively. Moreover, the best score obtained with respect to the varying number iterations is graphically represented in Fig. 5. Based on these results, it is analyzed that the proposed CSOM optimization technique provides an efficient results by finding the best optimal solution with reduced number of iterations. Due to the proper parameter setup and migration loop execution, the best optimal solution is effectively computed with increased convergence rate.

**Figure 6:**
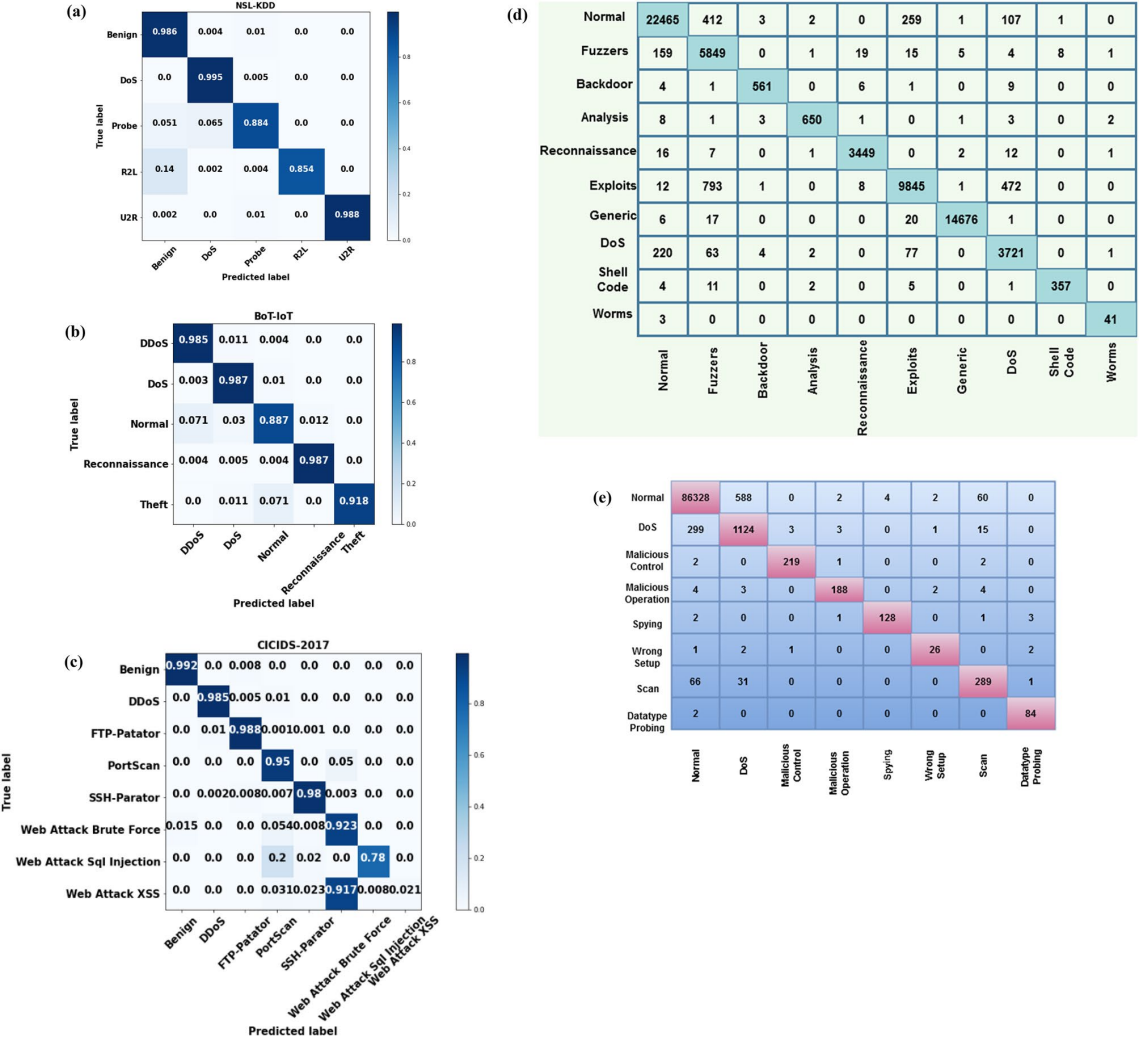
(**a**) Confusion matrix for NSL-KDD. (**b**) Confusion matrix for BoT-IoT IDS. (**c**) Confusion matrix for CICIDS 2017. (**d**) Confusion matrix for UNSW-NB 15. (**e**) Confusion matrix for DS2OS dataset.

Figure 6a to e presents the generated confusion matrix of the proposed Cyborg intelligence mechanism for the different types of datasets. Typically, the confusion matrix is mainly used to validate the detection performance of the classifier. According to the improved values of TPR, the increased accuracy of classifier is determined. In this analysis, the confusion matrices are validated for all types of cyber-threat datasets. The estimated results prove that the combination of proposed CSOM-RMML based Cyborg intelligence mechanism provides an accurate predicted results by properly detecting intrusions and its appropriate classes.

**Figure 7:**
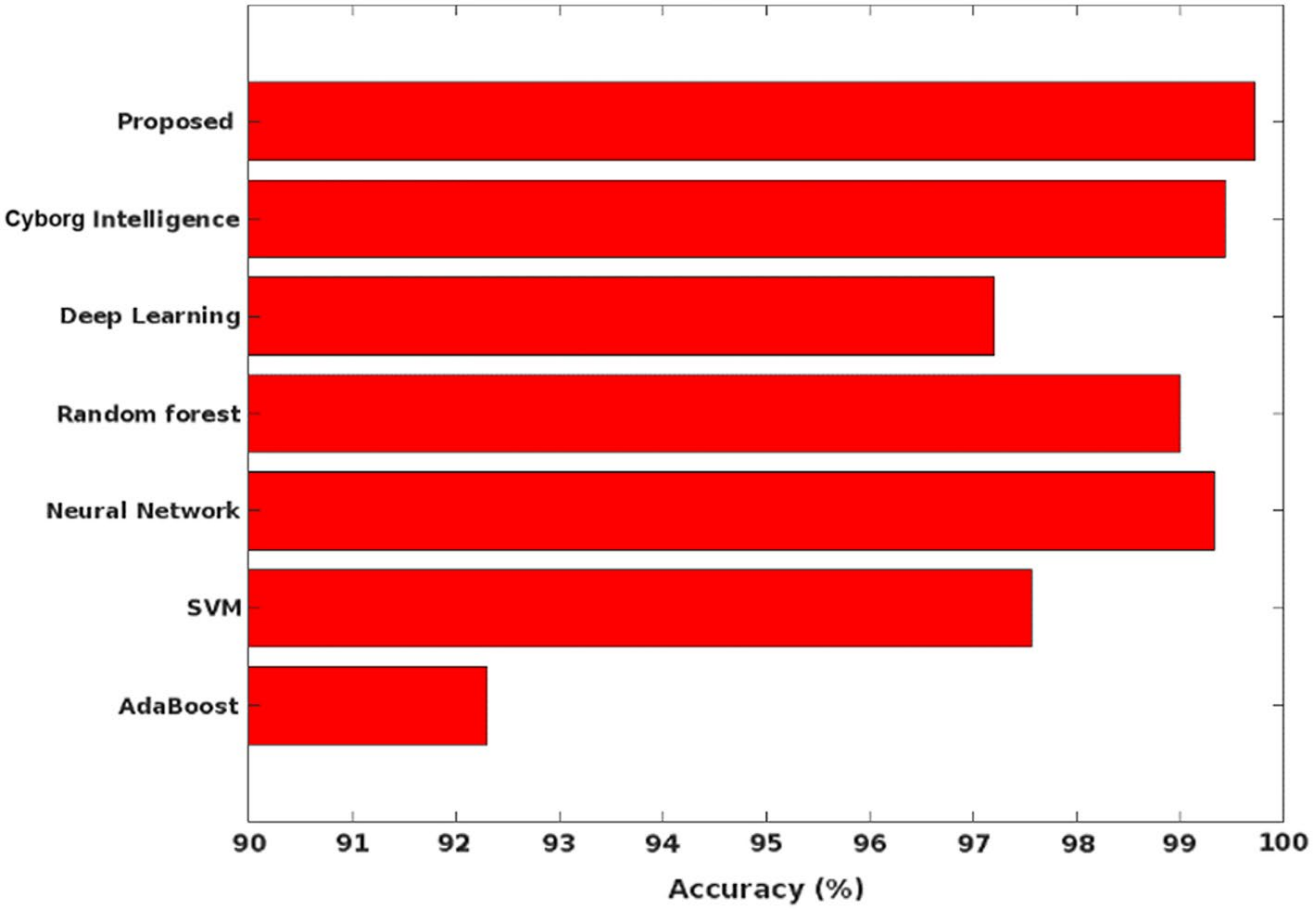
Accuracy analysis.

**Figure 8:**
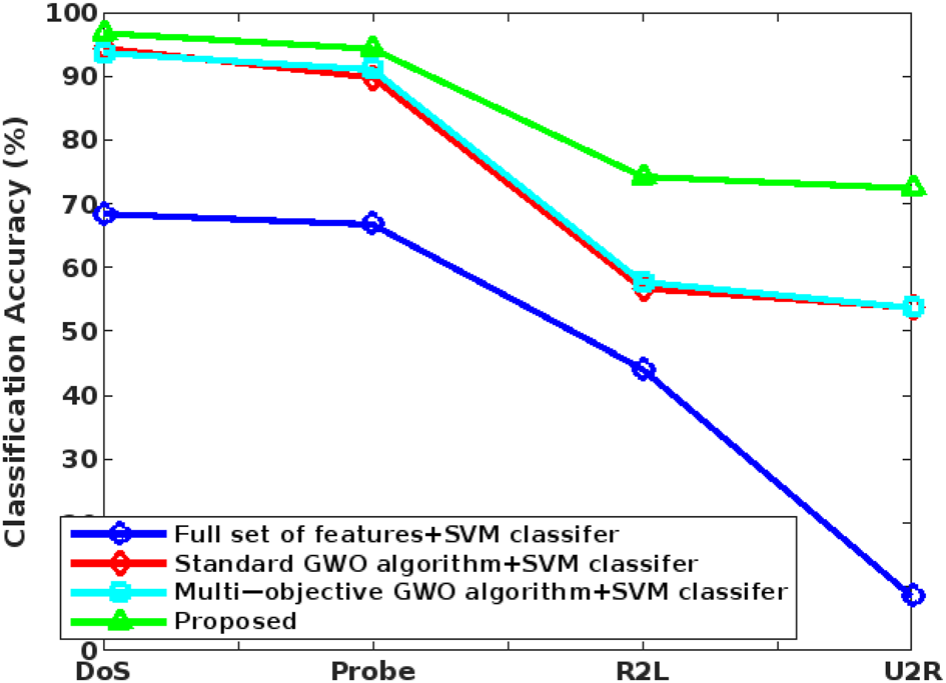
Classification accuracy for NSL-KDD.

The accuracy, precision, recall, detection rate, and F1-score are mainly used to validate the detection results of classifier, which are estimated as follows:

47 $$Detection \,rate=TPR= \frac{TP}{TP+FN}$$

48 $$Precision= \frac{TP}{FP+TP}$$

49 $$Recall= \frac{TP}{FN+TP}$$

50 $$F1-measure= 2*\frac{Precision \times Recall}{Precision+Recall}$$

51 $$Accuracy= \frac{TP+TN}{TP+FP+FN+TN},$$

where, TP – True Positives, TN – True Negatives, FP – False Positives, and FN – False Negatives. Among other parameters, the accuracy is considered as one of the key factor used to assess the detection efficiency of the classifier. It must be improved for ensuring the better system operations and performance. Figure 7 shows the accuracy of the conventional and proposed COSM-RMML attack detection approaches used for securing the smart city networks. The obtained results depict that the COSM -RMML technique overcomes the other approaches with increased accuracy. Similarly, the classification accuracy is estimated for the conventional^50^ and proposed optimization integrated classification techniques according to the different types of classes of NSL-KDD dataset in Fig. 8.

**Figure 9:**
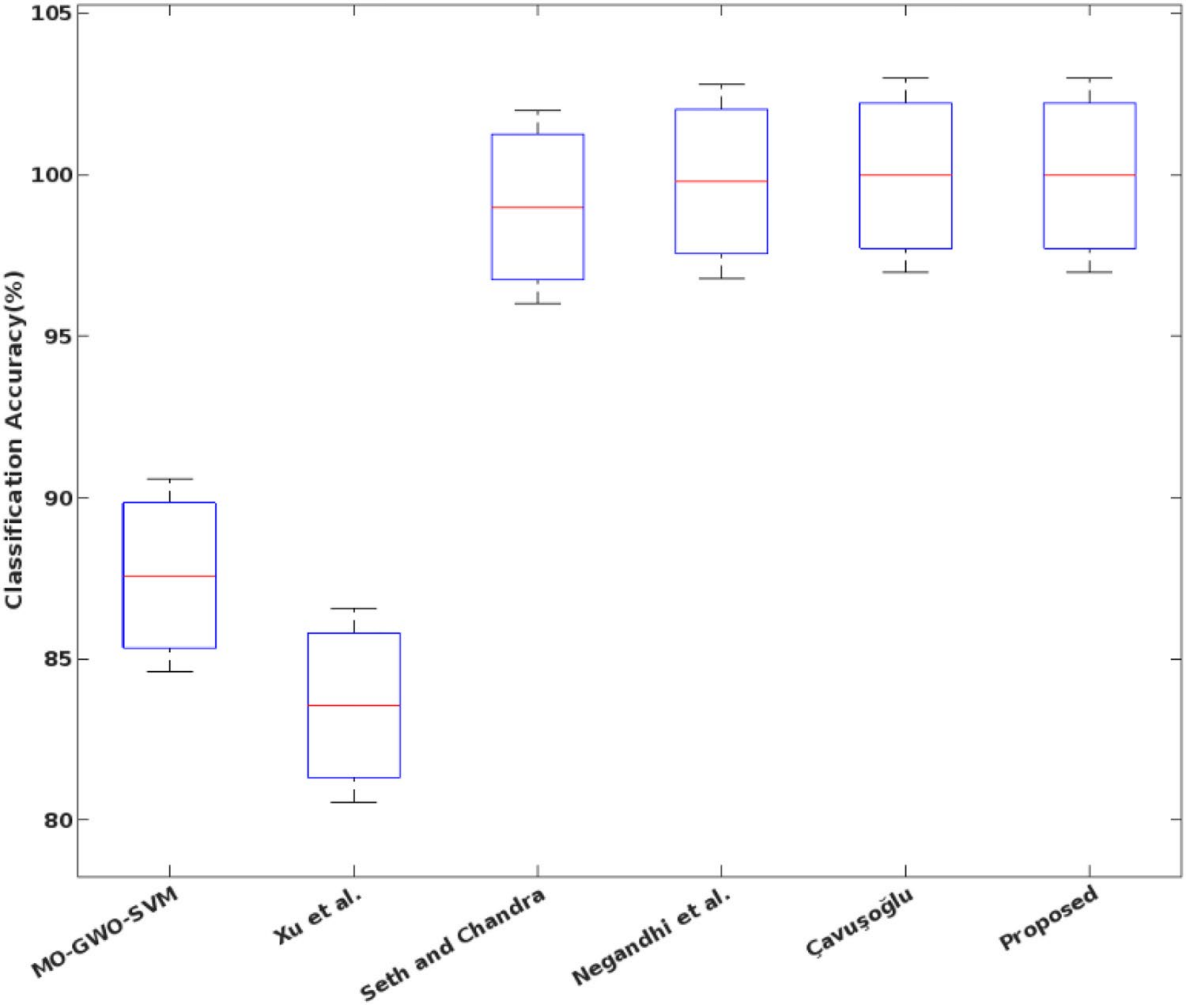
Accuracy of various optimization integrated classification technique.

**Figure 10:**
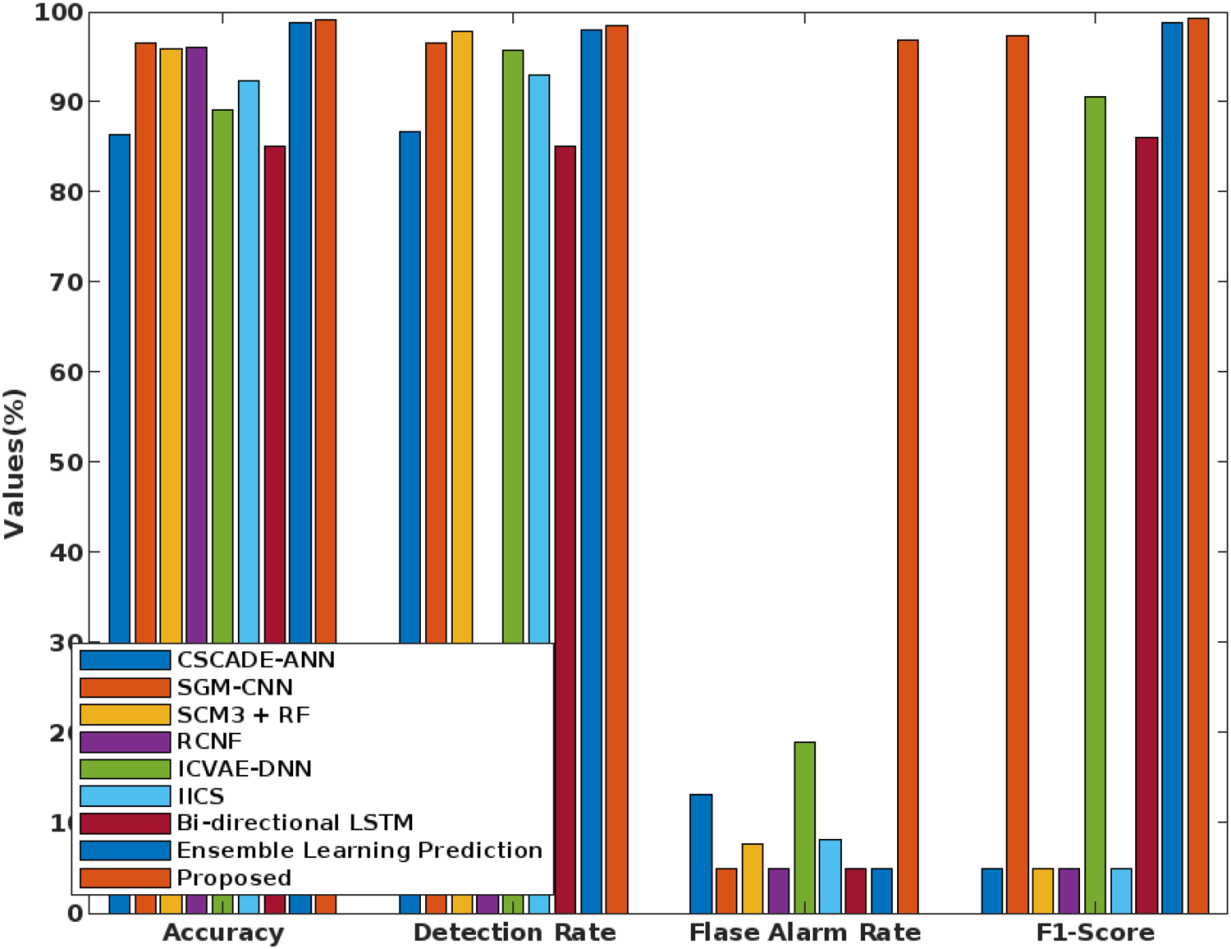
Overall performance analysis.

In addition to that, the overall accuracy value is validated for the multi-objective optimization based classification techniques by using the NSL-KDD dataset. Based on the computed results, it is clearly illustrate that the proposed COSM -RMML technique provides an increased accuracy for all types of attacking classes, which is highly improved than the conventional approaches. Due to the proper feature identification, the classifier training and testing operations are enhanced, which supports to obtain the maximum accuracy during intrusion detection and classification. Accuracy of various optimization integrated classification technique in represented in Fig. 9. Figure 10 presents the overall performance analysis of the conventional and proposed classification based intrusion detection approaches. Here, the results are estimated in terms of accuracy, detection rate, False Alarm Rate (FAR), and f1-score. According to the results, it is evident that the combination COSM -RMML technique overwhelms the other approaches with improved performance results.

**Figure 11:**
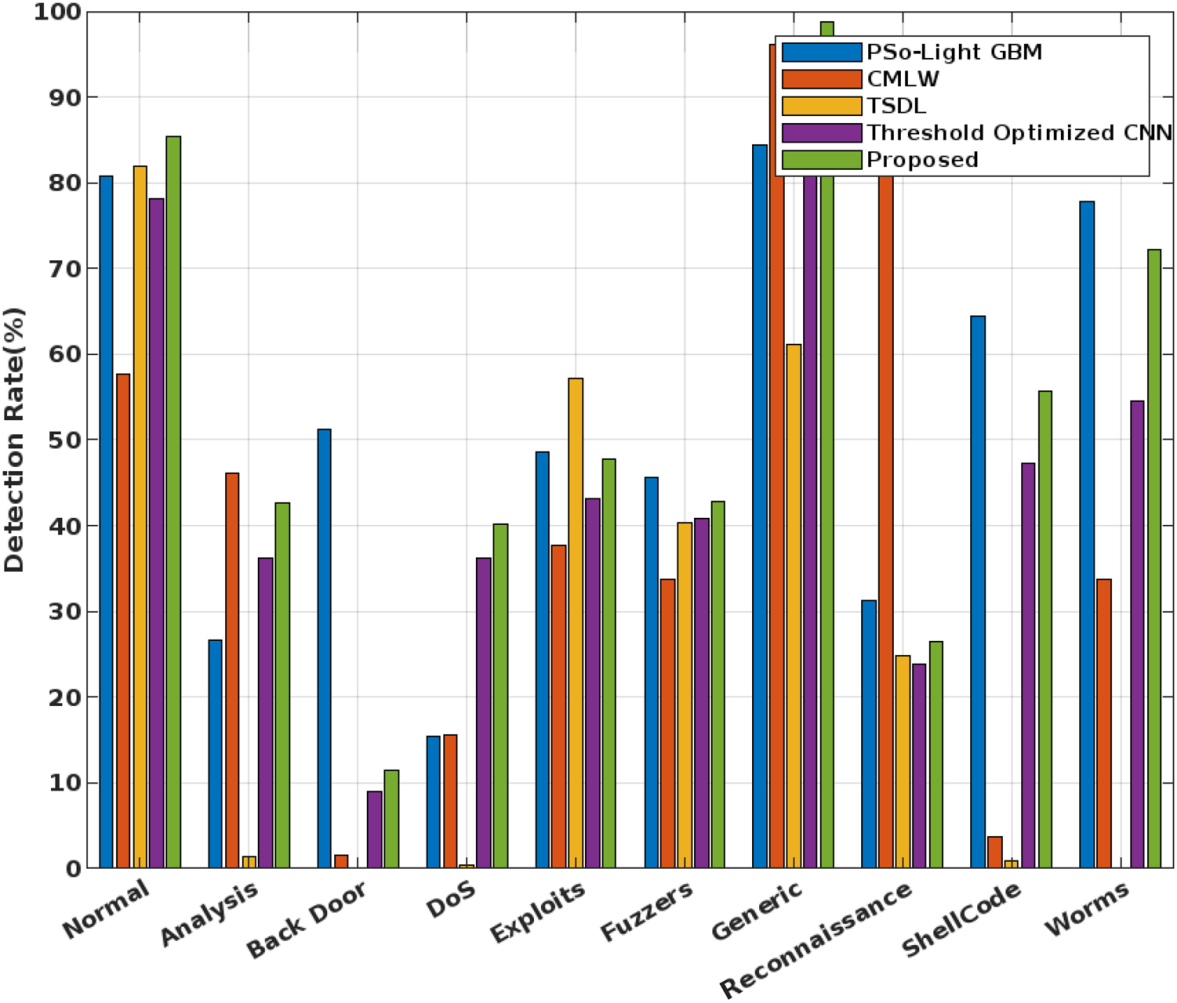
Detection rate using UNSW-NB 15 dataest.

**Figure 12:**
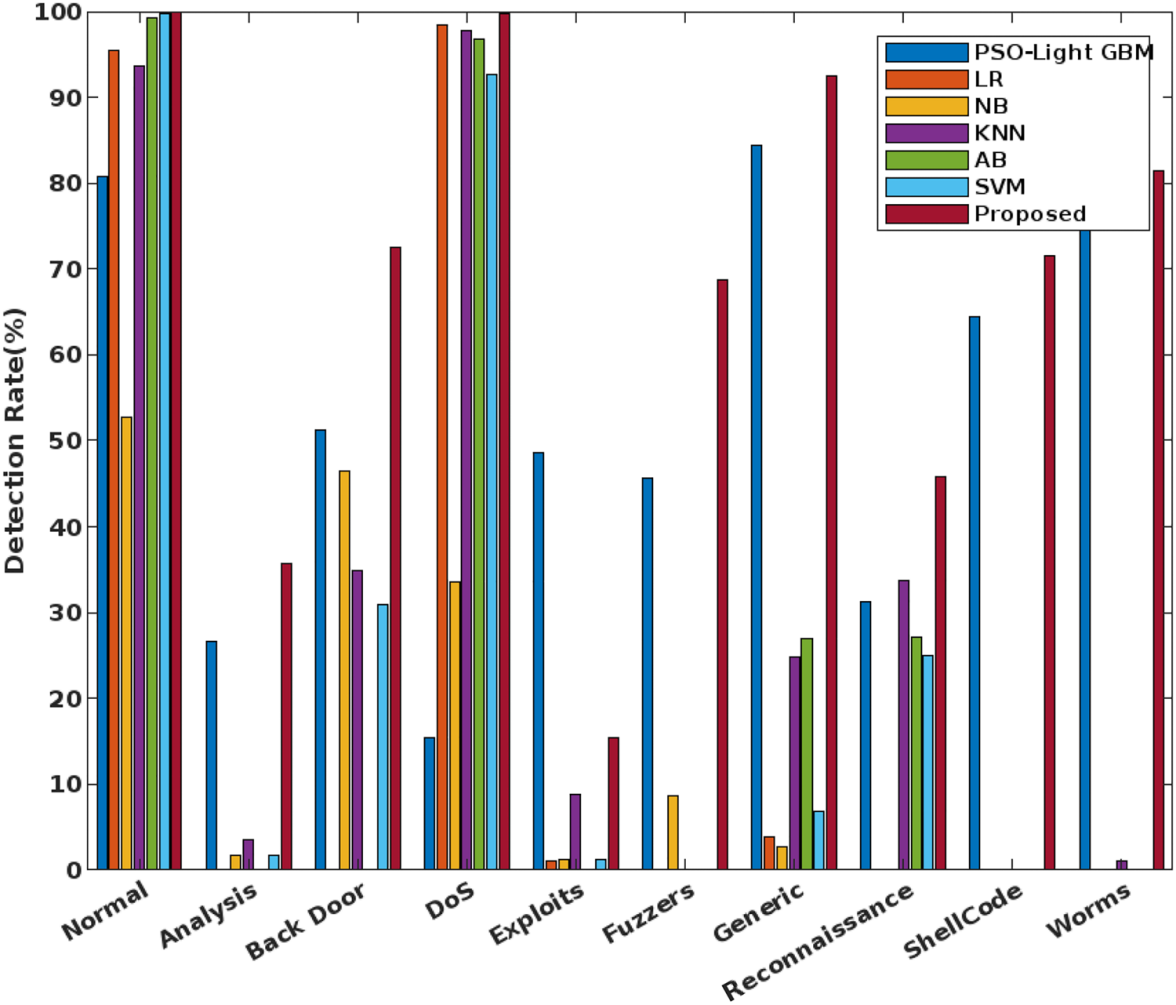
Detection rate of various machine learning techniques using UNSW-NB 15 dataset.

Consequently, the detection rate is validated for the state-of-the-art IDS mechanisms, and standard machine learning techniques^51^ as shown in Figs. 11 and 12 respectively. During this evaluation, the detection rate is assessed for the nine different types of attacking classes and normal class of the UNSW-NB15 dataset. Among other mechanisms, the proposed COSM-RMML technique has an excel detection rate for most of the attacking classes, specifically for the worms, shellcode, and generic cases. The proposed technique is highly robust and reliable, hence it has the strong detection ability in comparing the other classification approaches.

**Figure 13:**
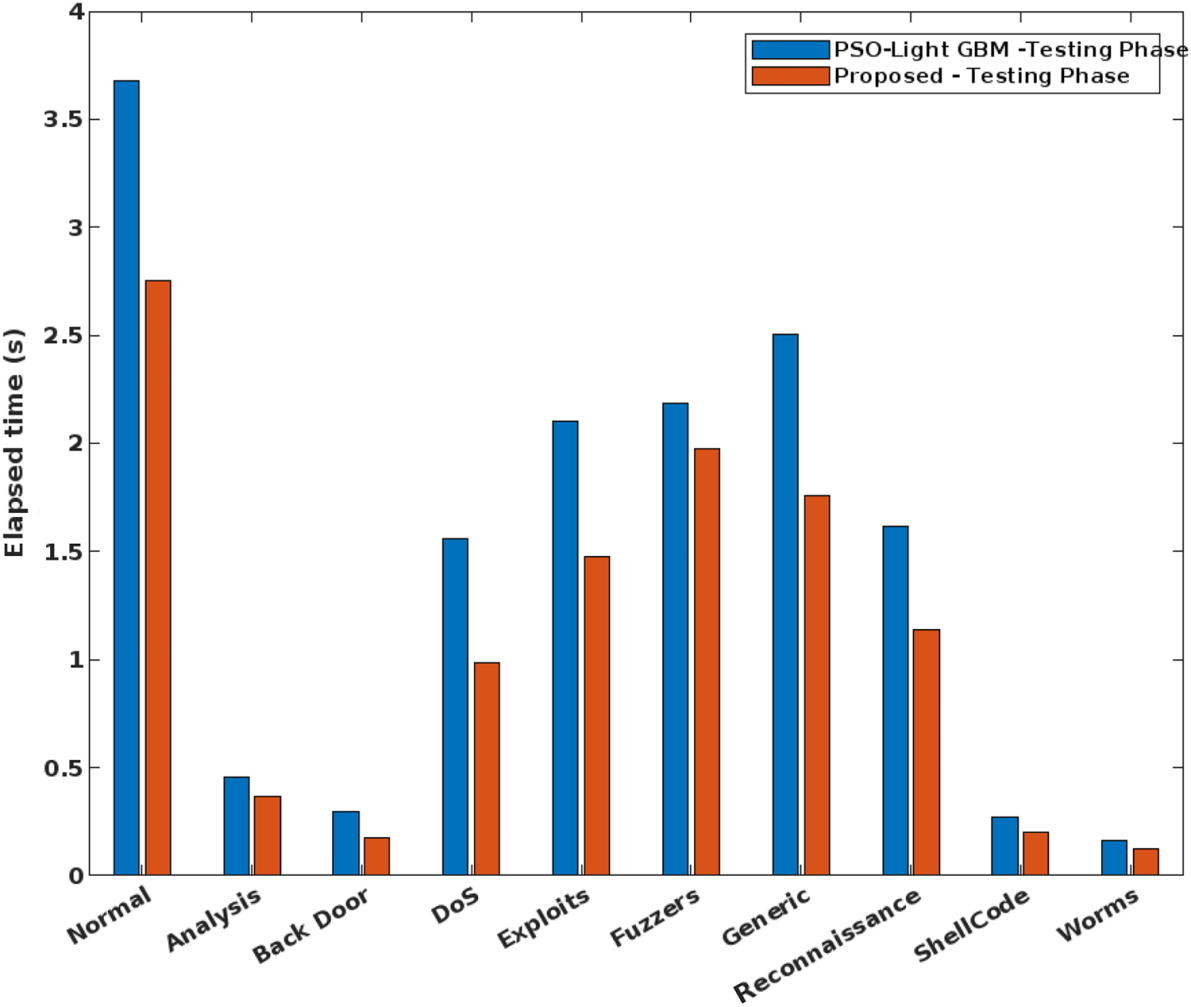
Elapsed time analysis using UNSW-NB 15 dataset.

**Figure 14:**
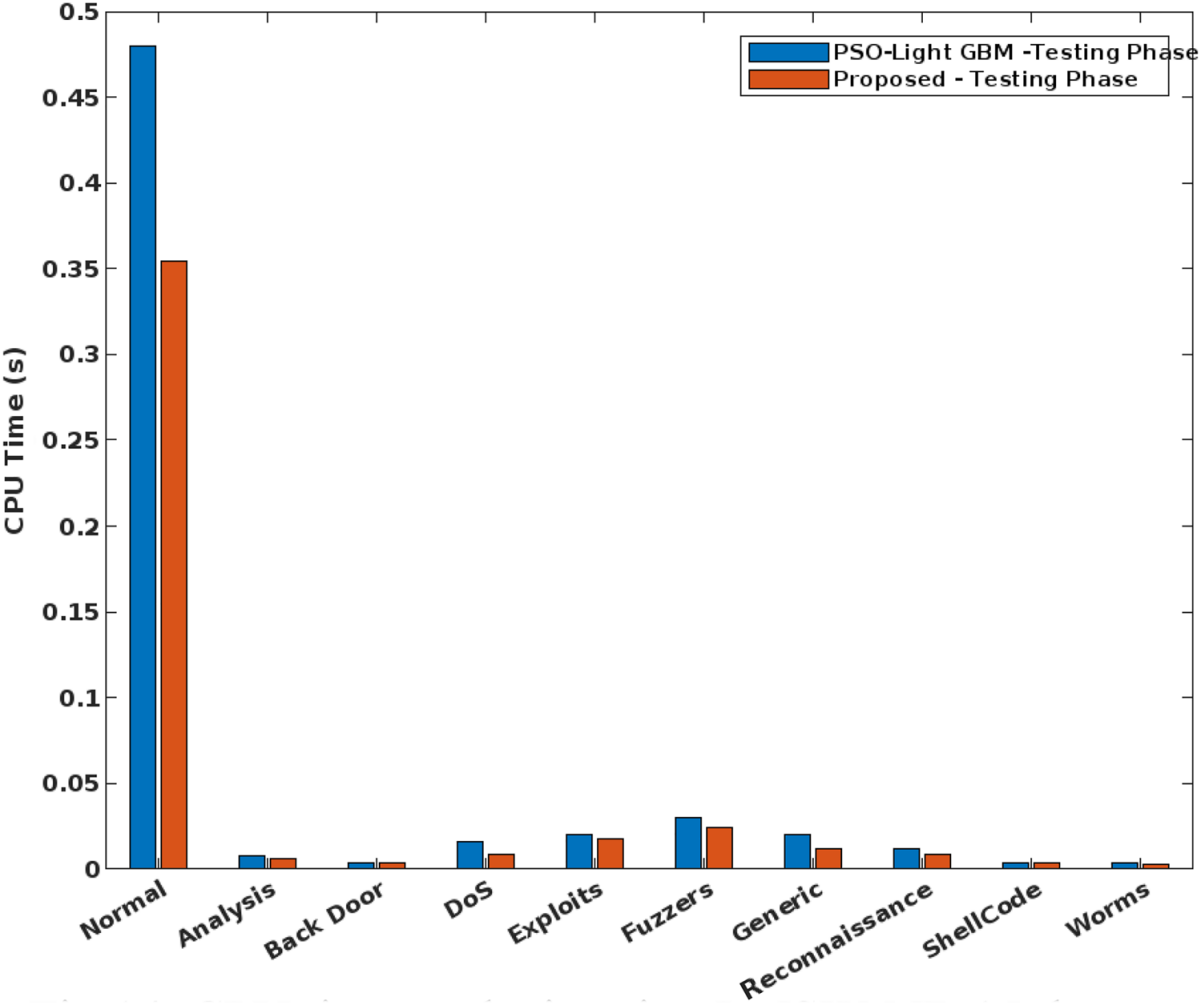
CPU time analysis using UNSW-NB 15 dataset.

**Figure 15:**
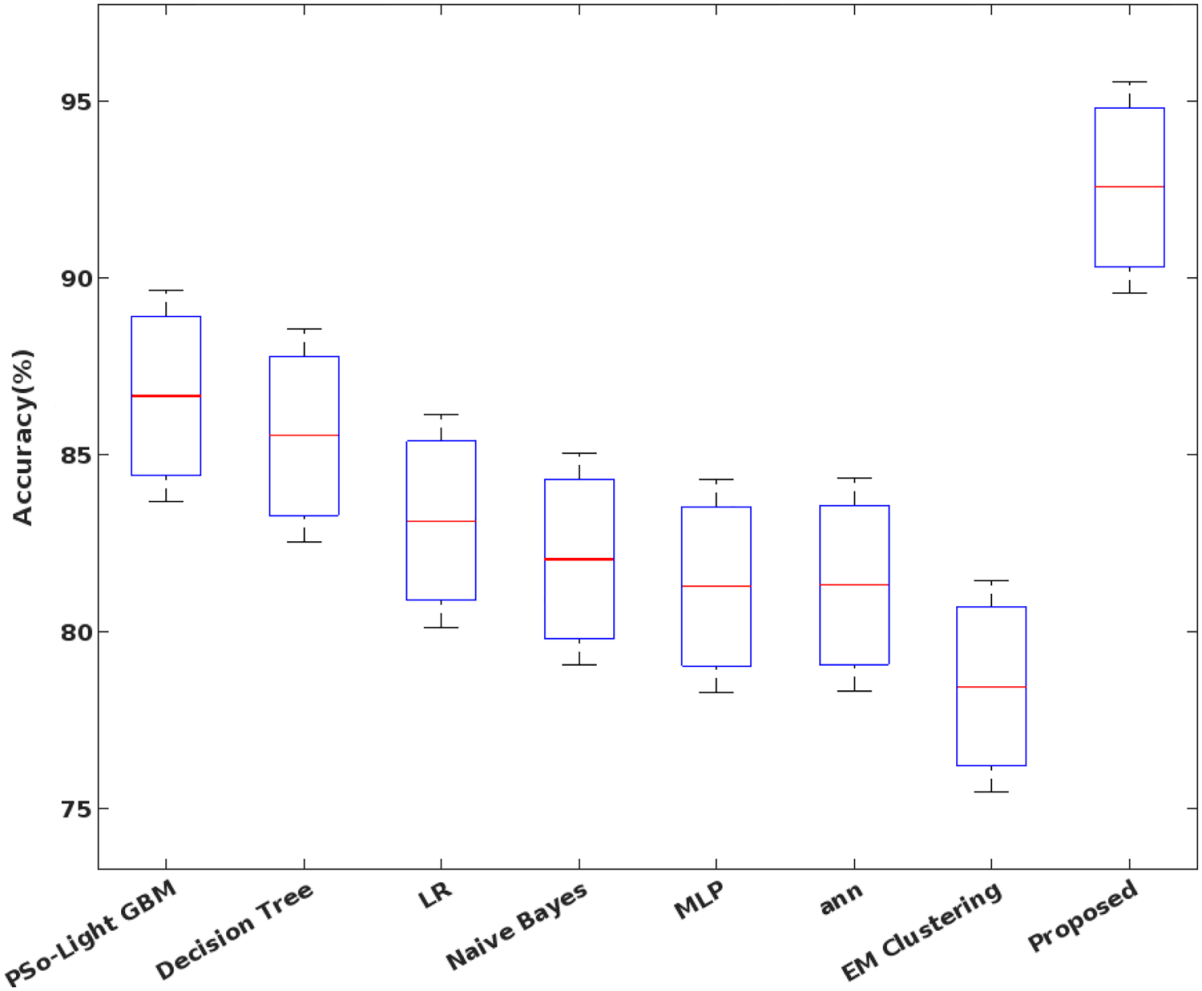
Accuracy of machine learning classifiers using UNSW-NB dataset.

In addition to that, the elapsed time and CPU time of the conventional and proposed security approaches are validated and compared in Figs. 13 and 14 respectively. Here, the time analysis is performed according to the different types of attacking classes in the UNSW-NB 15 dataset. Typically, the time cost can vary for both training and testing operations of classifier that is highly proportional to the type of predicted class. For instance, the normal class has the largest proportion during training and testing, hence it takes an increased amount of time with low frequency of data. From the observed results, it is identified that the proposed COSM-RMML technique requires the reduced time cost, when compared to the conventional approach. Moreover, the accuracy of the standard machine learning and proposed classification models are validated by using UNSW-NB 15 dataset as shown in Fig. 15.

**Figure 16:**
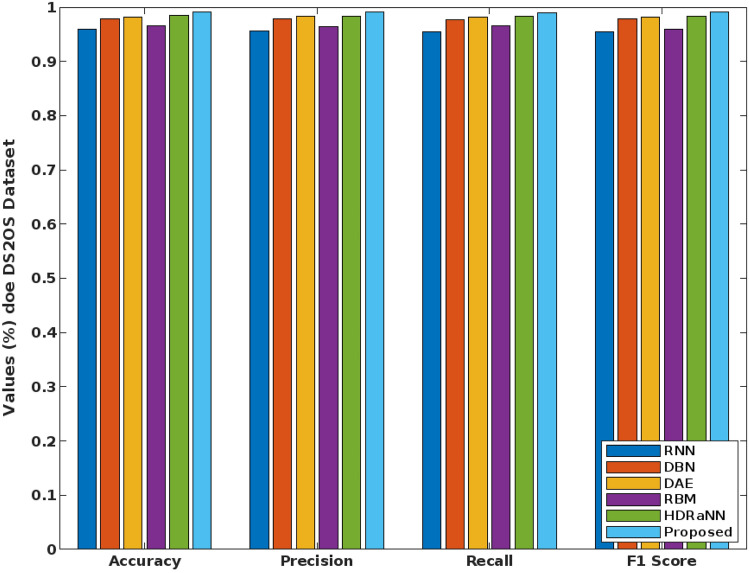
Performance analysis for DS2OS dataset.

**Figure 17:**
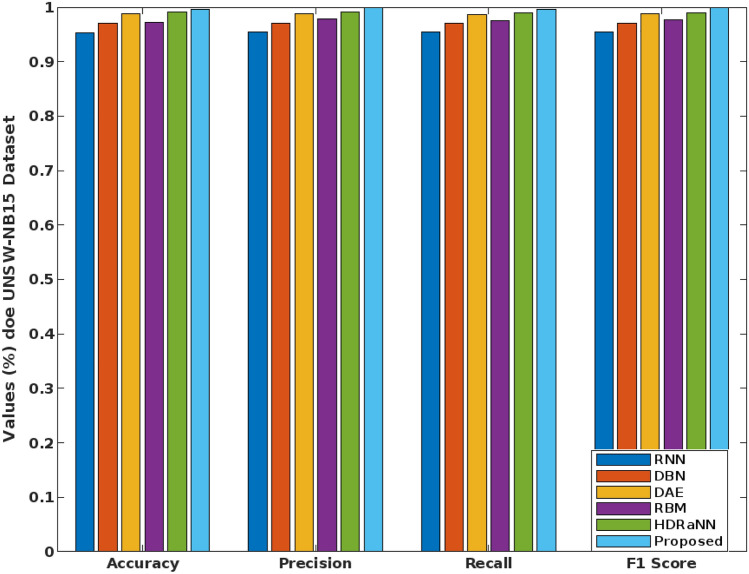
Performance analysis for UNSW-NB15 dataset.

**Figure 18:**
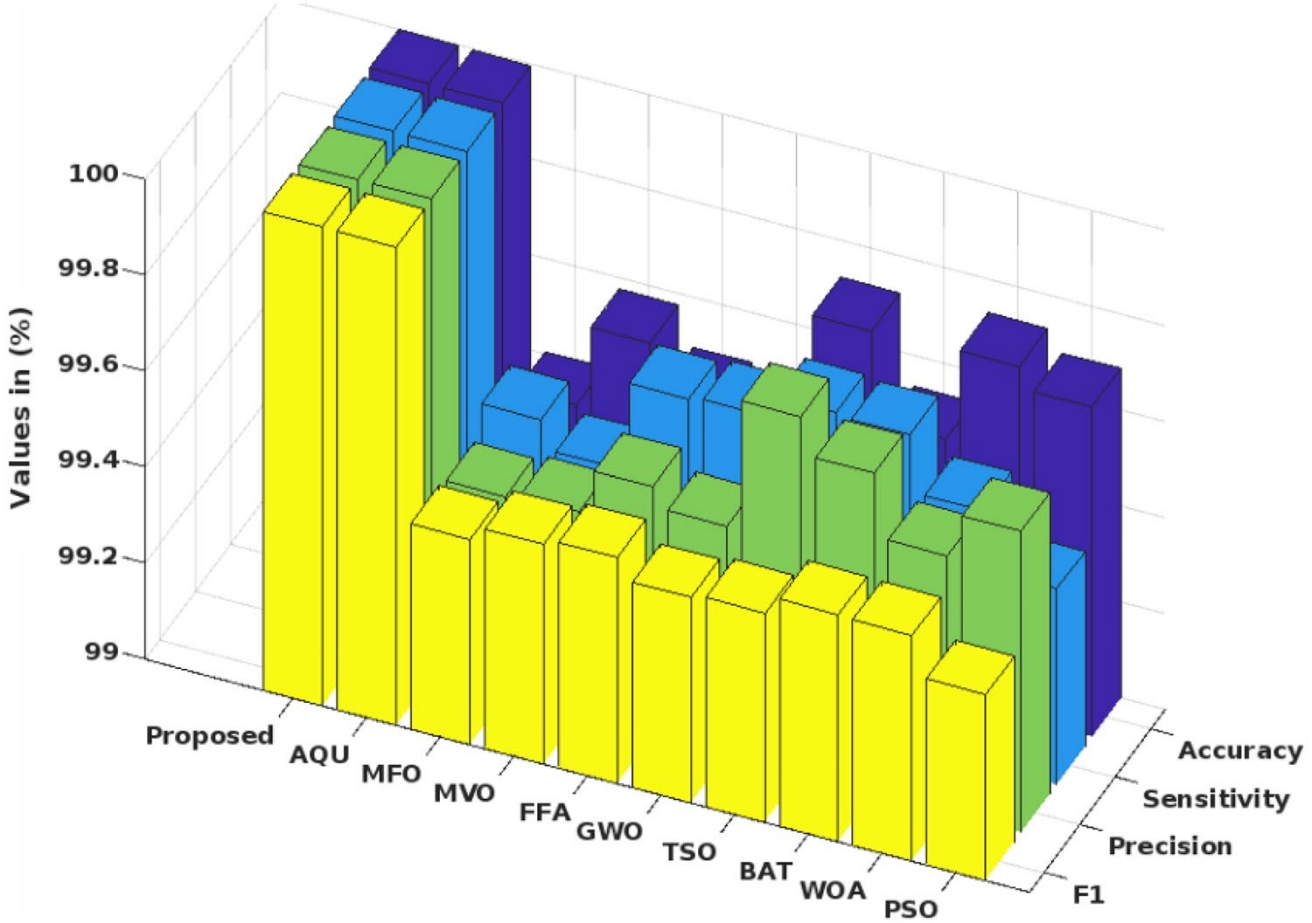
Performance analysis of CICIDS 2017 dataset.

Similarly, the overall performance results of the conventional and proposed COSM-RMML intrusion detection approaches are validated and compared by using DS2OS, UNSW-NB15, and CICIDS-2017 dataset as represented in Figs. 16, 17 and 18. Here, the results are estimated in terms of accuracy, precision, recall, and f1-score. For proving the superiority, the proposed security framework is validated and tested by using these DS2OS, UNSW-NB15 and CICIDS 2017 datasets. Depending on the type of attacking classes, the detection rate and accuracy of classifier can vary. From these results, it is evident that the combination of COSM-RMML has an increased capability to handle all kinds of datasets with improved performance outcomes. When compared to the other approaches, the results are highly increased in the COSM-RMML system, which illustrates the superiority and betterment of the proposed model.

**Figure 19:**
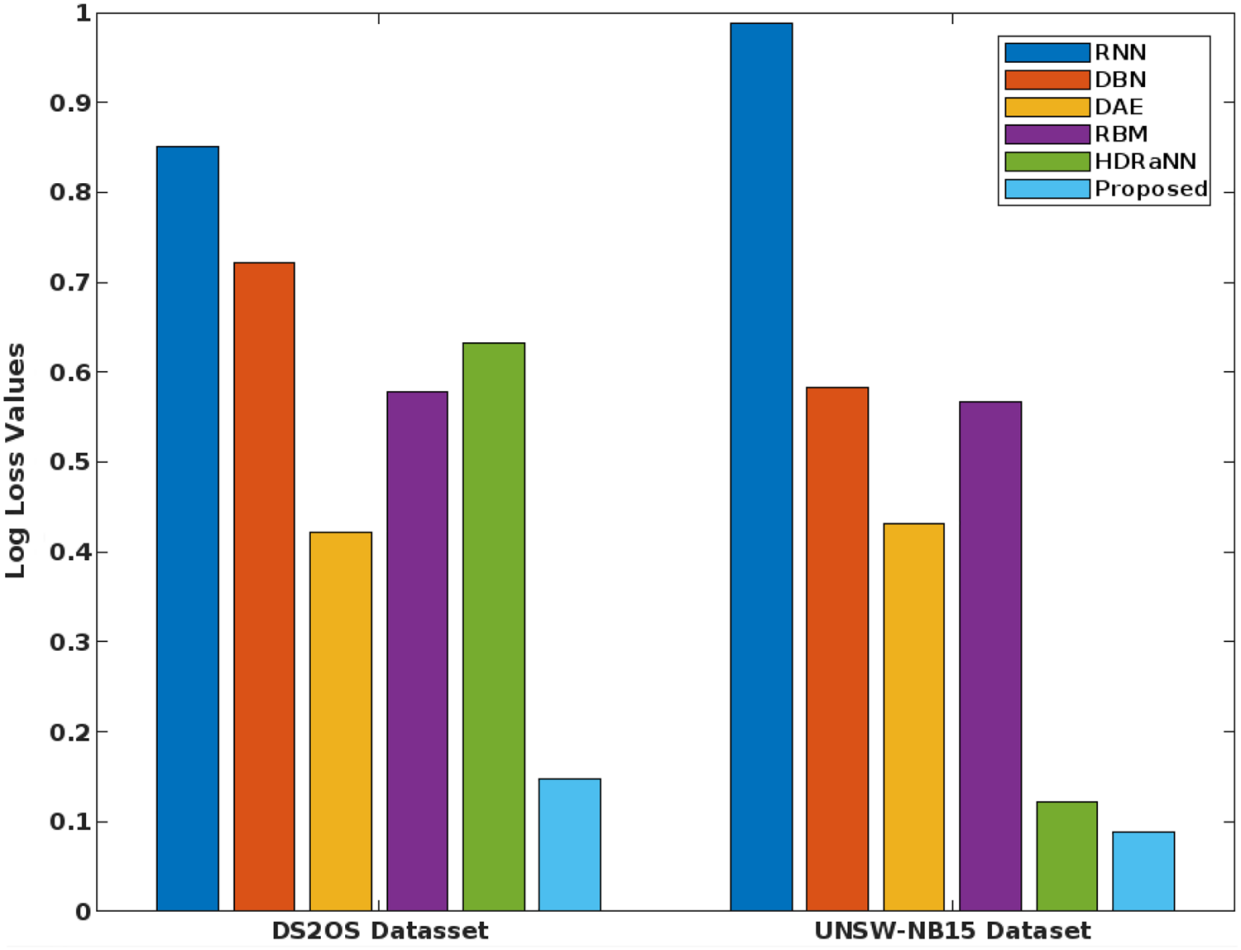
Log loss value.

**Figure 20:**
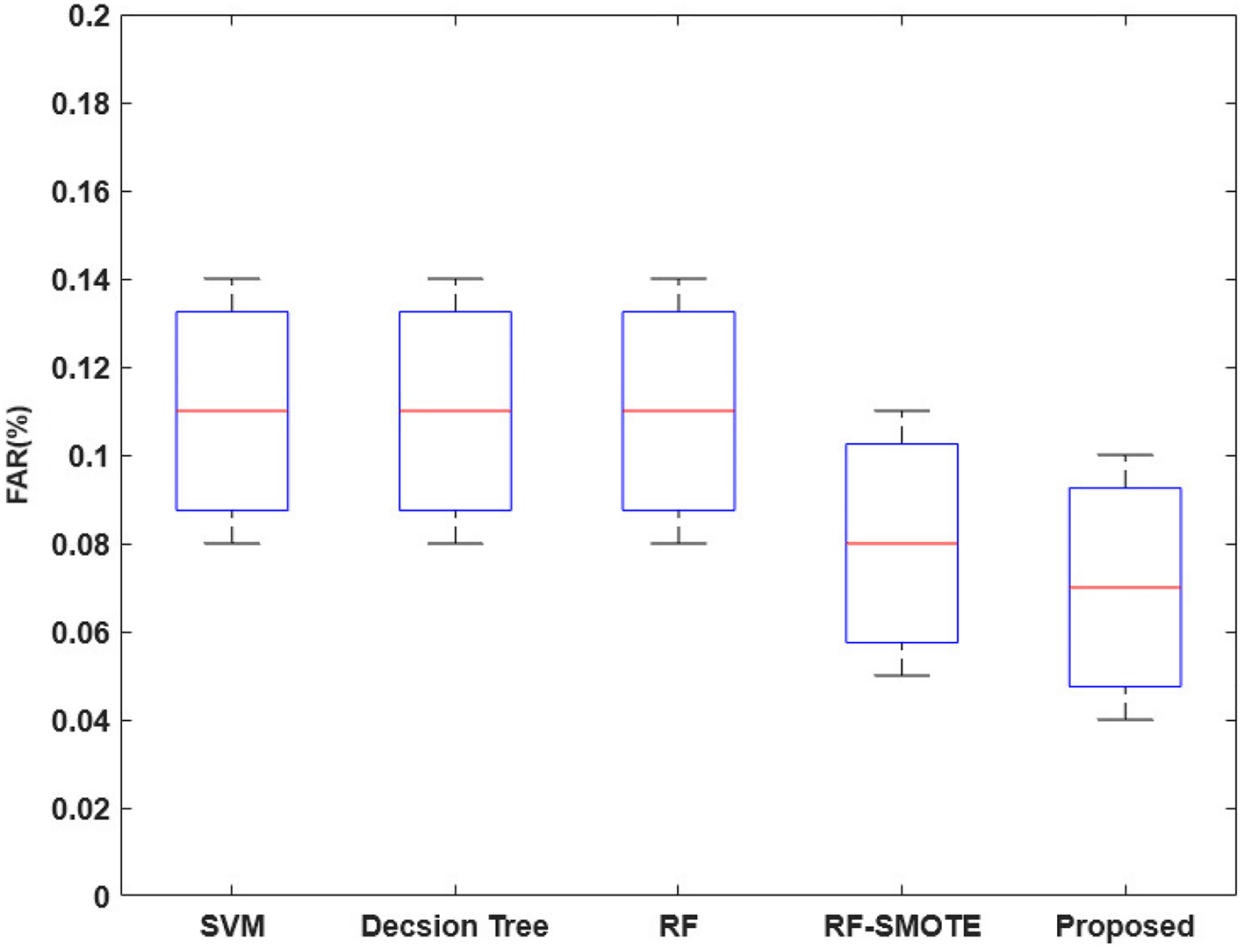
FAR of BoT-IoT IDS dataset.

Figure 19 validates the log loss value of the existing and proposed classification techniques for both DS2OS and UNSW-NB-15 datasets. Typically, the log loss value should be minimized for ensuring an accurate detection operations, because the increased loss value can degrade the performance of entire security model. Based on the estimated analysis, it is observed that the proposed COSM-RMML technique provides the reduced log loss value for both datasets by properly handing the input datasets. Furthermore, the FAR of the standard machine learning and proposed techniques are validated and compared by using the BoT-IoT IDS dataset as shown in Fig. 20. Due to the proper training and testing of features in the classifier, the FAR of the proposed classifier is effectively reduced, when compared to the other approaches.

In this study, several parameters including accuracy, detection rate, false alarm rate, f1-score and time consumption have been estimated for assessing the performance of the proposed model. For this validation, the distinct and more popular intrusion datasets are used in this work, which helps to evaluate the performance results of the proposed model. For the NSL-KDD dataset, the intrusion classification accuracy is increased to 99% with respect to the different types of attacks in the dataset. Similarly, the detection rate is improved up to 99.5% for the UNSW-NB 15 dataset with the accuracy of 99.6%. Moreover, the elapsed time is reduced to 0.2 s in the proposed system by using UNSW-NB 15 dataset.

## Conclusion

In order to safeguard the networks of smart cities against cyber threats, this article introduces a new security paradigm based on cyborg intelligence. This work's key contribution is the creation of a low-complexity computational and economical intrusion detection framework for smart city security. Here, this security approach is put into practice using the most well-known and widely accessible benchmark datasets. The stages of data pretreatment and imputation, feature optimization, intrusion detection, and categorization are all included in this framework. In the beginning, the QIDI technique is used to carry out the data imputation and normalization procedures, where the identification of the missing fields and the removal of undesired attributes are carried out to provide the filtered data. The preprocessed dataset's characteristics are then extracted using the best optimal solution offered by the COSM mechanism. This optimization involves the parameter initialization, formation of individual populations, convergence testing, refinement, and identification of the best optimal solution processes. Following feature selection, the cutting-edge RMML technique is used to anticipate and classify the intrusion in accordance with the chosen features. With less training and testing time, our classifier predicts and categorizes the type of cyber-threat. In this work, various cyber-threat datasets, including the UNSW-NB 15, NSL-KDD, BoT-IoT IDS, DS2OS, and NSL-KDD, are used to test security systems. Additionally, the accuracy, precision, FAR, f1-score, log loss, elapsed time, and CPU time of the findings are validated. The effectiveness of the suggested systems is then proven by comparing the obtained findings to cutting-edge approaches and traditional machine learning techniques. This analysis comes to the conclusion that the COSM-RMML technique surpasses the competition, producing good performance outcomes for all types of datasets. By using the proposed model, the classification accuracy for all the datasets used in this work is maximized up to 99.2% with the detection rate of 99% and low time consumption of 0.2s. In future, the present work can be further enhanced by deploying the transfer learning model for maximizing the security of IoT enabled smart city networks with low cost consumption.

## Data Availability

The data that support the findings of this study are available from the corresponding author, upon reasonable request.
